# Sensory adaptations reshaped intrinsic factors underlying morphological diversification in bats

**DOI:** 10.1186/s12915-021-01022-3

**Published:** 2021-04-30

**Authors:** J. H. Arbour, A. A. Curtis, S. E. Santana

**Affiliations:** 1 Present Address: Department of Biology, Middle Tennessee State University, Murfreesboro, TN 37132 USA; 2 Department of Biology, University of Washington, Seattle, Washington 98195 USA; 3 Burke Museum of Natural History and Culture, University of Washington, Seattle, Washington 98195 USA

**Keywords:** Modularity, Allometry, Comparative phylogenetics, Geometric morphometrics, Echolocation, Skull

## Abstract

**Background:**

Morphological evolution may be impacted by both intrinsic (developmental, constructional, physiological) and extrinsic (ecological opportunity and release) factors, but can intrinsic factors be altered by adaptive evolution and, if so, do they constrain or facilitate the subsequent diversification of biological form? Bats underwent deep adaptive divergences in skull shape as they evolved different sensory modes; here we investigate the potential impact of this process on two intrinsic factors that underlie morphological variation across organisms, allometry, and modularity.

**Results:**

We use comparative phylogenetic and morphometric approaches to examine patterns of evolutionary allometry and modularity across a 3D geometric morphometric dataset spanning all major bat clades. We show that allometric relationships diverge between echolocators and visually oriented non-echolocators and that the evolution of nasal echolocation reshaped the modularity of the bat cranium.

**Conclusions:**

Shifts in allometry and modularity may have significant consequences on the diversification of anatomical structures, as observed in the bat skull.

## Background

Extrinsic and intrinsic factors enhance or limit morphological evolution, thereby determining how lineages move towards adaptive optima. While access to novel resources (e.g., habitats, prey) can create opportunities for macroevolutionary trait shifts and morphological diversification [1, 2], scaling, developmental, constructional, and functional principles ultimately determine the range of morphologies that can evolve towards or within new adaptive zones [3–6]. For example, mechanical trade-offs between power and velocity in the vertebrate lower jaw may limit evolution towards extreme jaw mechanical advantage [3–5], and the strength of such functional constraints has been correlated with the rate of diversification of relevant morphological traits in some fish lineages [4]. Connecting intrinsic factors with patterns of adaptive evolution can therefore reveal the relative contribution of ecological and organismal processes to morphological diversity. Advances in phylogenetic comparative methods and methods for the analysis of highly multidimensional shape data have opened new opportunities to document discontinuities in the macroevolutionary trends of complex biological form [7–11] and test hypotheses regrading what factors impact the diversification of morphology.

Allometry and modularity are two intrinsic factors that structure morphological variation and can constrain or promote trait diversification [12–15]. Allometry describes disproportionate changes in biological shape with organismal size [16]. While the commonly studied ontogenetic allometry describes shape change with growth over an organism’s life, size-shape relationships can be identified at similar age classes across species [17–19]. These trends in “evolutionary allometry” may be conserved across highly disparate clades—for example, a pattern of rostrum elongation with larger body size (termed “cranial evolutionary allometry”, CREA) is found across numerous mammalian orders [20–22]. Allometry may offer a “line of least evolutionary resistance” [15] that biases pathways of evolutionary shape change. However, lineages experiencing evolutionary allometry might become constrained in the use of size-mediated ecological resources [19], or exhibit mismatches between performance and realized niches [23].

Modularity describes the partitioning of covariation among traits into separate “modules” [12, 14, 24], or groups of anatomical traits that show strong correlations with one another, but weak correlations with traits outside the module [12, 25, 26]. Modules may be defined intra-specifically (ontogenetic or static modularity) or inter-specifically (evolutionary modularity) [12], and traits within modules may be linked by functional properties, developmental origins, genetic basis, or mutual selective constraints [25]. Modularity imposes unequal organization of trait covariation, impacting morphological diversity, and the potential for anatomical structures to adapt to different functions. For example, differences in trait integration between anterior and posterior limb modules are believed to have contributed to differences in limb and locomotor diversity between placental and marsupial mammals [27–29]. While much research has focused on understanding the underpinnings of morphological modules, their macroevolutionary consequences have been less frequently addressed [13, 25, 30], and there is no consensus on the impact of integration and modularity on morphological diversification or disparity [12, 14, 30–33].

Here, we test whether adaptive morphological evolution has been impacted by changes in skull allometry or modularity across an exceptionally diverse mammal group, Chiroptera (bats). Bats are one of the largest mammalian Orders (1400+ species) [34], span nearly one order of magnitude in skull length, and their skull shapes range widely across axes of elongation, flexion, width, and height [35, 36]. Skull morphological diversity in bats includes other extreme morphological features such as bulbous rostral inflations, dish-like facial shields, moveable premaxillae, non-pathological cleft palates, flanges on zygomatic arches, and non-existent or massive sagittal crests [37–39]. This broad anatomical spectrum within the relatively simple mammalian skull template provides a uniquely well-suited system to examine how trait covariation changes within and among anatomical parts, and with size, across a whole clade.

**Fig. 1.**
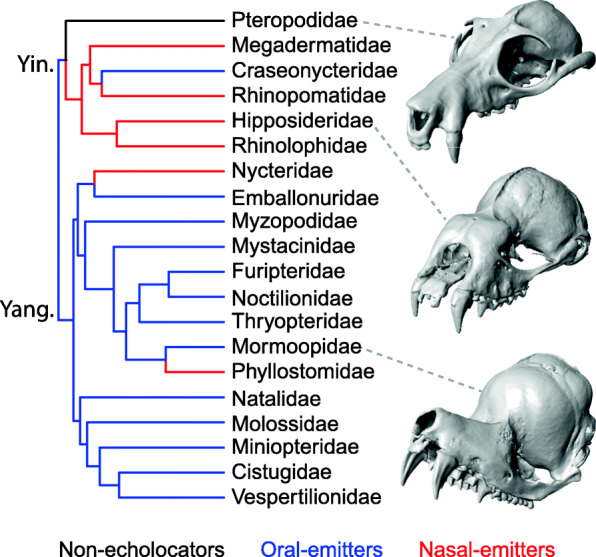
A hypothesized evolutionary history of echolocation among bat families. Oral emission has been supported as the ancestral state by previous molecular and fossil studies [40, 41], but there remains uncertainty on this character state. Skull images show representatives of each echolocation type: from the top—*Pteropus poliocephalus, Hipposideros caffer,* and *Mormoops blainvillei*. Yin = Yinpterochiroptera, Yan = Yangochiroptera

A recent analysis of skull shape evolution across Chiroptera revealed adaptive shifts associated with modes of echolocation sound emission (i.e., nasal vs. oral emission, loss of echolocation), which strongly impacted early morphological divergence and modern skull shape disparity [35]. However, this impact was uneven across skull components; echolocation-associated shifts drove divergence along the major axes of cranial but not mandibular shape variation. Here, we test the hypothesis that evolutionary transitions in echolocation mode (Fig. 1) led to cranial morphological divergence via modification of intrinsic factors (allometry, modularity) that determine cranial shape in bats. First, we predict that bats exhibit an allometric pattern of rostral elongation with skull size as in other mammals [20], but expect the strength of this relationship to vary between visually oriented non-echolocators versus oral/nasal emitters because echolocating bats are under strong size constraints imposed by the physics of high-frequency echolocation call production [40] and the rostrum has additional functional constraints in nasal emitters (below). Second, we predict that the evolution of nasal echolocation reshaped the modularity of the bat cranium as the rostrum took on the new role in sound transmission [37]. As this largely shifted the function of echolocation call production from the mouth (i.e., rostrum plus mandible) to only the cranium, we also predict that evolutionary shifts in modularity are decoupled between the cranium and mandible in nasal emitters.

We used geometric morphometrics, comparative phylogenetic and cluster analyses to (1) assess patterns of allometry and detect morphological modules in the skull across Chiroptera and within echolocation modes, (2) contrast results with previous hypotheses of bat/mammalian skull modularity [37, 42–44], and (3) test whether different regions of the bat skull differ in evolutionary lability. In doing so, we provide the first quantitative links between adaptive shifts and macroevolutionary patterns in skull shape evolution and underlying intrinsic factors across bats.

## Results

### Morphological and allometric variation across echolocation modes

**Fig. 2.**
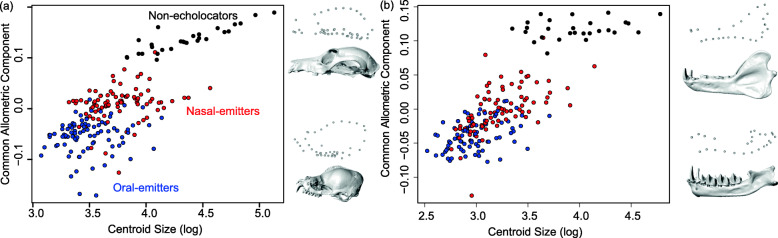
Allometry of the bat cranium (**a**) and mandible (**b**) across 202 and 191 bat species, respectively. Shape variation with allometry is plotted as the score of the common allometric component vs. the log of centroid size for each structure. Landmark configurations show the calculated shape of the skull and mandible, respectively, in lateral view based on allometric trends at the largest and smallest centroid size in each dataset. Skull images provide examples of species with some of the largest and smallest crania and mandibles; crania: *Pteropus vampyrus* (top) and *Craseonycteris thonglongyai* (bottom); mandibles: *Pteropus poliocephalus* (top) and *Craseonycteris thonglongyai* (bottom)

Without phylogenetic correction, there is a significant relationship between skull size and shape across bats (cranium and mandible *p* = 0.001; *N* = 202 species), with size explaining a moderate amount of shape variation (*R*^2^: cranium = 18.3%, mandible = 22.3%). However, allometric analyses of cranial and mandible shape showed both a difference in slope (test of common allometries: cranium F = 4.01, df = 198, *p* = 0.01, mandible F = 3.59, df = 185, *p* = 0.01) and in the variation explained by size between echolocators and non-echolocators (Fig. 2). Shape variation was more weakly explained by differences in size among echolocators compared to non-echolocators across both the cranium and the mandible (Table 1). Larger cranial size was associated with a more elongate rostrum in non-echolocating bats (Additional file 1: Figure S6) [45–51], in contrast with dorsoventral expansion of the rostrum in oral emitters, and complex shape changes involving the profile of the rostrum, the curvature of the zygomatic arches, and the positioning of the teeth and premaxilla in nasal emitters (Fig. S6). Compared to the cranium, differences in allometric trends in mandible shape were subtler among echolocation modes. While mandible shape differed between the three modes examined, with non-echolocators showing a more ventrally oriented mandibular body and a taller coronoid, larger mandibles were consistently associated with more robust morphologies (e.g., taller body of the mandible) across all three echolocation groups (Fig. S6).

**Table 1:** Allometric trends in cranial and mandibular shape across bats (including echolocation as a covariate) and each echolocator group individually. Results of non-phylogenetically informed analyses are presented

	***R***^**2**^	***F***	***P*** value
**Cranium**			
**Across echolocators**			
Log centroid size	0.183	73.0	0.001
Echolocator type	0.299	59.6	0.001
Size × echolocator	0.0252	5.01	0.001
**Within echolocators**			
Non-echolocators	0.261	9.19	0.001
Oral Emitters	0.0396	3.55	0.003
Nasal Emitters	0.103	9.63	0.001
**Mandible**			
**Across echolocators**			
Log centroid size	0.225	71.2	0.001
Echolocator type	0.168	26.7	0.001
Size × echolocator	0.0225	3.56	0.003
**Within echolocators**			
Non-echolocators	0.229	6.97	0.001
Oral emitters	0.0866	7.87	0.001
Nasal emitters	0.132	11.7	0.001

When phylogenetic correction was applied, the relationship between cranial size and shape remained significant, but size explained very little shape variation across bat skulls (both *p* = 0.001, cranium *R*^2^ = 6.21%, mandible *R*^2^ = 10.3%). The amount of shape variation explained also differed between laryngeal echolocators (*R*^2^: cranium–oral emitters = 4.40%, nasal emitters = 6.23%; mandible–oral emitters = 11.5%, nasal emitters = 10.6%) and non-echolocators (*R*^2^ cranium = 27.3%, mandible = 22.9%). Overall, evolutionary allometry had a greater impact on cranial and mandibular shape variation in pteropodid non-echolocators than echolocating bats in all other families.

### Modularity of the bat cranium

**Fig. 3.**
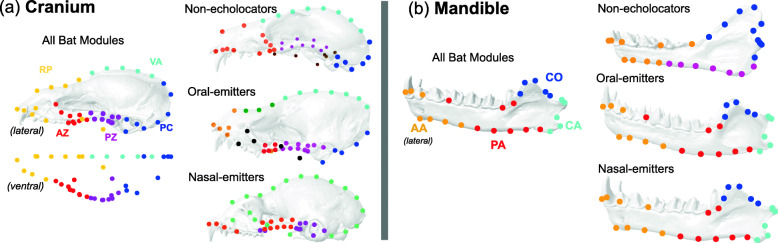
Detected modules of bat crania (**a**) and mandibles (**b**) based on cluster analysis of a phylogenetic congruence matrix (see Fig. S7–8). Left: Across all bats, the modules detected across the cranium were: RP—rostrum/palate/pterygoid, VA—vault, AZ—anterior zygomatic arch, PZ—posterior zygomatic arch, PC—posterior cranium encompassing the basicranium, auditory bulla and the intersection of the lambdoidal and sagittal crests. Right: Across all bats, the modules detected across the mandible were: AA—anterior alveolus region, PA—posterior alveolus region, CO—coronoid process, CA—condyle and angular process. Morphological modules are also shown for each of the echolocator types. Landmarks show the consensus configuration in lateral view within each dataset, and representative crania and mandibles have been warped to match these configurations

Across 202 bat species, five cranial modules were supported in an eigen analysis of the phylogenetic congruence matrix from the Procrustes superimposed landmark coordinates. A cluster analysis of the congruence matrix estimated the five modules as follows: (1) rostrum (and palate + pterygoid hamulus) (RP), (2) anterior zygomatic arch (AZ), (3) posterior zygomatic arch (PZ), (4) midline of the cranial vault (VA), and (5) posterior cranium encompassing the basicranium, auditory bulla and posterior sagittal crest/intersection of the sagittal and lambdoidal crests (PC) (Fig. 3 and S7). This cluster analysis linked the modules from the braincase and posterior zygomatic (3–5) and the rostrum and anterior zygomatic (1–2) (Additional file 1: Fig. S7). The phylogenetically corrected covariance ratio (CR) statistic significantly supported these five modules, as did EMMLi analyses with separate within- and between-module covariances, over hypotheses involving no modules, six modules [42], and a face-braincase two module system [52, 53] (Table 1).

When echolocator groups were analyzed separately, their number of modules differed from that detected across all bats (Fig. 3), being greater among oral emitters (7) and lower among nasal emitters (3). Cranial modules in oral emitters showed the strongest parallels to those observed across all bats, in terms of the groupings of specific landmarks into modules and the covariation between landmarks (Additional file 1: Fig. S7). Oral emitters possess the VA, PC, and PZ modules seen across all bats, but show divisions within the RP and AZ modules (Fig. 3). Comparatively, nasal emitters grouped landmarks from the RP, VA, and PC into one large module, and non-echolocators grouped the AZ and RP landmarks, while also possessing a separate “basicranial” module (Fig. 3). Additionally, the CR showed no significant modularity among the detected modules within each emission mode, but did indicate significant modularity using the five “all bat” modules within oral emitters alone (Table 2). Both non-echolocators and oral emitters maintained a divide between the face and braincase modules, whereas the majority of the cranium was represented by one module encompassing elements from the face and braincase in nasal emitters (Fig. 3).

**Table 2:** Results of phylogenetic covariance ratio analysis of cranial and mandibular modularity. *indicates statistical significance after Holm-Bonferroni correction

		Cranium			Mandible		
	Modules	# of Mods	CR	***p***	# of Mods	CR	***p***
**All bats**	All Bats	5	0.9662	0.005*	4	1.1700	1
**Non-echolocators**	All Bats	5	1.1291	1	4	1.0687	0.419
	Non-Echolocators	5	1.1578	1	3	1.1545	1
**Nasal emitters**	All Bats	5	1.0299	0.461	4	1.1925	1
	Nasal emitters	3	1.1228	1	4	1.1883	1
**Oral emitters**	All Bats	5	0.8742	0.001*	4	1.0952	0.620
	Oral emitters	7	1.0829	0.655	4	1.0773	0.566

Results from a phylogenetic CR approach [24, 54] provided support for the “all bat” modules, both across all 202 species as well as within oral emitters alone, but not within nasal emitters or non-echolocators. Comparatively, EMMLi analyses indicate that the best supported modules were those detected for each of the echolocator types specifically (e.g., oral emitter modules for oral emitters; Table S4). Within-module correlations were generally higher in modules associated with the cranial vault, zygomatic arches, and elements of the rostrum, but the strength of the relationships between parts of the cranium was quite variable (Fig. S9). Overall, CR and EMMLi both found strong support for significant morphological modules across all bats and across oral emitters specifically.

### Modularity of the bat mandible

Eigen- and cluster analysis of mandible phylogenetic congruence coefficients identified four modules across 191 species (Fig. 3). Following the nomenclature of morphological modules used by Monteiro and Nogueira [43], mandibular modules included two modules representing the body of the mandible, the (1) anterior alveolar region (including the symphysis, incisors, canines and premolars) and (2) posterior alveolar region, including the molars, (3) mandibular condyle plus angular process, and (4) coronoid process. These were very similar to the modules used in analyses of the phyllostomid mandible by Monteiro and Nogueira [43], with the exception of the joint angular and condyle module (Fig. 3).

Mandibular modules in both oral and nasal-emitting bats showed very strong similarity to those found across all bats (Fig. 3 and S8), with minor differences involving the division between the anterior and posterior alveolar regions. Like the cranium, the morphological variation in oral emitting bats was the most reflective of patterns found across all bats (Fig. 3 and S8–9). In contrast, detection of modules in non-echolocators showed a different partitioning of mandible morphological variation, with three modules combining the posterior alveolar region and the angular process, and the coronoid process and mandibular condyle. EMMLi did not support non-echolocator-specific modules, but rather found support for the Monteiro-Nogueira [43] modules, which similarly divide the angular regions and condyle as described above (Table S5). Regardless, both detection and EMMLi results suggest a significant departure between non-echolocator modularity and that observed within echolocating bat species (Fig. 3 and Table 2; Additional file 1: Fig. S9 and Table S5). Overall support for mandibular modularity varied between EMMLi and phylogenetic CR, with the phylogenetic CR approach showing poor support for modularity in the mandible (Table 2 and S5).

### Heterogeneity in shape evolution across the bat skull

**Fig. 4.**
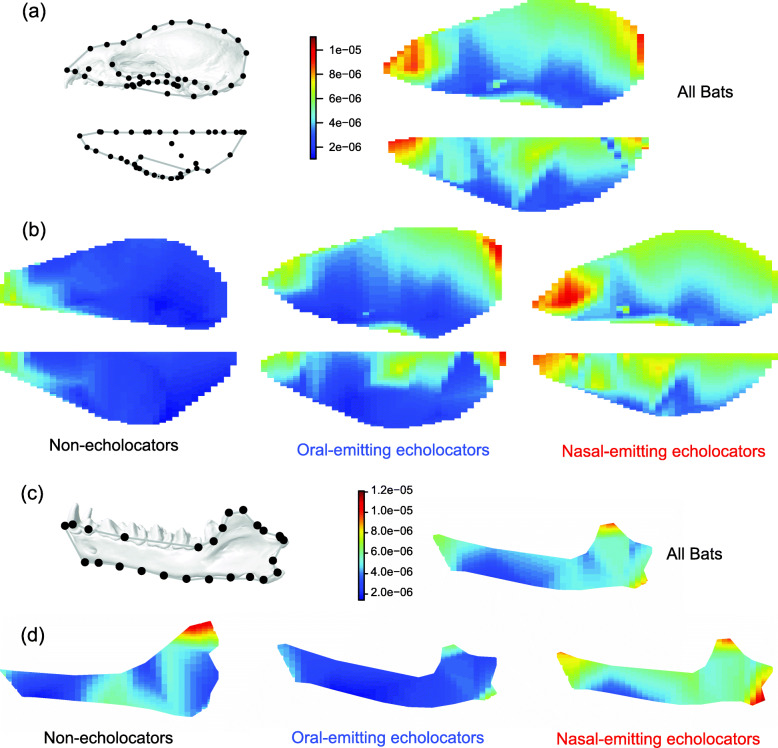
Brownian motion evolutionary rates (σ^2^) across the cranium (**a, b**) and mandible (**c, d**) in bats. Values are evolutionary rates at landmark locations and linearly interpolated rates between landmarks. The cranium is shown in lateral (top) and dorsal (bottom) view for each pair. The mandible is shown in lateral view. Rates were calculated across all bats (**a**, **c**) as well as within each of the echolocation groups (**b**, **d**). Landmarks show the consensus configuration in lateral view within each dataset and representative crania and mandibles have been warped to match these configurations

The rates of morphological evolution varied across the skull of bats based on both Q- and R-mode approaches; however, there was no link between patterns of evolutionary rates and morphological modules (Additional file 1: Table S6–7, “all bats”). Increased evolutionary lability was focused towards the anterior-most landmarks on the rostrum as well as the vault of the cranium, particularly at the intersection of the sagittal and lambdoidal crests (Fig. 4). On the mandible, high evolutionary rates were concentrated in the coronoid process, angular process, and the mandibular symphysis. Non-echolocators also showed a region of higher rates between the body and ramus of the mandible.

Both Q- and R-mode approaches showed strong support for evolutionary rate heterogeneity across both the cranium and the mandible for the all bat dataset, within oral emitters, and across the mandible of non-echolocators (Additional file 1: Table S5–6). There was mixed support for anatomical evolutionary rate heterogeneity for nasal emitters, which may be due to differences in the construction of the constraint matrix (see methods and Additional file 1: Fig. S3). However, nasal emitters showed faster rates across the entire cranium and mandible when compared to other bats (Fig. 4). Non-echolocators exhibited the slowest evolutionary rates across the cranium, and oral emitters showed very slow overall evolution of mandible shape (Fig. 4).

## Discussion

### Bat skull modularity

Our analyses of bat skull shape found support for shifts in both modularity and allometry in tandem with the evolution of echolocation modes in Chiroptera. Therefore, this study lends strong support to the hypothesis that adaptive shifts in mode of echolocation, the primary sense used by most bats, are associated with changes in intrinsic factors known to drive skull morphological diversity. The modules detected across the bat skull differ from some previous studies of mammals, such as the six-module placental mammal model (which did not sample bats [42];). While bats possess modules representing the anterior and posterior portions of the cranium—similar to the “vault” and “basicranium” in the six-module placental mammal model [42]—the 5-module bat model proposed here splits the anterior and posterior portions of the zygomatic into separate modules. This may reflect the osteology of the zygomatic arch in bats. The zygomatic arch is comprised of the jugal anteriorly and the squamosal posteriorly in most mammals, but the jugal is greatly reduced and the anterior zygomatic arch is comprised of an extension of the maxilla in bats [55]. The bat 5-module system also unites the rostrum (including the dentigerous region and palate) with the pterygoid hamulus, while the 6-module system divides the nasal, orbit, and molar regions, and links the pterygoid hamulus and portions of the zygoma [42]. Cranial modules in bats overall do not appear to be strongly linked to development origins, as at least one module (the “vault”) derives from both neural crest and mesoderm cell lines [56]. Additionally, both the bat 5-module model and the mammal 6-module system showed greater support in EMMLi than a face-braincase divide that would largely separate regions derived from the neural crest and mesoderm, respectively (Additional file 1: Fig. S4 and Table S3).

The 4-module system of the bat mandible presented here showed strong similarities to prior models proposed for mammals and phyllostomid bats [43]. Unlike some of these previous studies, however, these modules were detected from the geometric morphometric data itself, rather than pre-supposed based on mandibular development. Despite this, the four modules still match general patterns of morphogenesis in the mammalian mandible, as cell clusters forming the alveolar region and processes (coronoid, condyle, and angular) show an early developmental divide [57–59]. The “developmental history” hypothesis of mammal mandible modularity, as described by Zelditch et al. [58], predicts stronger integration between the angular process and condyle compared to the coronoid. Consistent with this, our analyses grouped the angular, “masseter” or ramus, and condyle within one module both across bats and within oral and nasal emitters. It is intriguing that the cranium and mandible at least qualitatively appear to be differentially driven by developmental modularity, considering they must articulate during growth and development. However, here we have not directly tested the relative strengths of developmental patterns between the two datasets, and the patterns of mandible modularity may yet be driven by non-developmental factors. The mandible showed fewer changes in patterns of modularity within bats, with a significant shift only in non-echolocating bats when cluster analysis was used. However, EMMLi found support for a similar divide between the mandible body and mandibular processes (Additional file 1: Fig. S9).

The patterns of modularity found in oral-emitting bat crania likely reflect those ancestral to bats. Some previous reconstructions of ecological, anatomical, and sensory traits have suggested that the ancestor of modern bats was a small-bodied, oral echolocating insectivore [40], and there is generally strong support for the ancestor of modern bats having possessed laryngeal echolocation. However, the evolutionary history of echolocation among early bat lineages remains controversial due to the sparse fossil record of stem bat lineages and stem pteropodids [40, 60, 61]. Presuming an oral-emitting ancestor, the evolution of other echolocation modes, including the evolution of nasal emission and the loss of echolocation, were associated with shifts in modularity as we predicted. Consistent with novel functional demands on the nasal cavity for sound transmission and modification, the repeated evolution of nasal emission in bats was associated with restructuring of cranial, but not mandibular modularity. Modularity of the cranium was reduced among nasal-emitting bats, compared to the presumed ancestral state among bats and to oral emitters and non-echolocators. Previous modularity analyses of Phyllostomidae [33] and Rhinolophidae [37] have also found that these nasal-emitting families possess more integrated crania when compared to oral emitters.

We also found that mandible shape, and the shape of regions of the cranium interfacing with the mandible, evolve more slowly in oral emitters. In contrast, we found overall faster mandible shape evolution in both nasal emitters and non-echolocators, potentially reflecting lower constraints on the mandible due to its lack of or lower involvement in echolocation in these groups. It is also possible that the dorsoflexion of the rostrum associated with oral emission [35] imposes more severe constructional constraints on mandible shape in oral emitters, compared to nasal emitters and non-echolocators, which possess more ventriflexed rostra. While the ancestral bat was likely an insectivore [40], a state that most oral emitters have retained (albeit a few species are carnivorous), both nasal emitters and non-echolocators have diversified into other feeding roles (e.g., frugivory, nectarivory, sanguivory, omnivory). This may be associated with the higher evolutionary rate of the mandible in the latter two groups.

### Non-echolocators revert to ancestral mammalian cranial allometry

Diversification of skull morphology in non-echolocating bats appears to have been driven primarily by a shift in cranial and mandibular allometry. Size was most strongly linked to shape variation in non-echolocators than in echolocating bats. Non-echolocating bats encompass the largest bat species (e.g., up to 1.7 m wingspan in *Acerodon jubatus*), and their renewed reliance on vision for navigation and foraging likely released constraints on body size while also imposing different pressures on the size and morphology of the cranium.

Non-echolocating bats rely on vision for navigation, possess relatively larger eyes than echolocating bats [40], and can have partial to nearly complete post-orbital bars (comprising processes from the frontal and zygoma) similar to those seen in other large-eyed mammal groups—a trait which is absent in echolocating bats. Thus, pressures to accommodate the eyes and their support structures likely imposed different constructional constraints on the skull of non-echolocators when compared to other bats [6]. Indeed, the dominant allometric pattern in non-echolocators consists of lengthening the rostrum anterior to the orbit. This pattern of skull elongation parallels mammal CREA (cranial evolutionary allometry), in which smaller species have short faces while larger species have longer faces [20, 21]. As the ancestor of bats likely was an oral echolocator [40, 41], this suggests that non-echolocators reverted to a CREA ancestral pattern while echolocating bats evolved less typical patterns of skull allometry (Fig. S5).

It is important to note that our analyses only address changes in evolutionary allometry—i.e., relationships between size and shape across multiple species. While oral and nasal emitters do not show a strong relationship between size and shape across species, at least in one major clade of nasal emitters (Phyllostomidae), shifts in ontogenetic allometry within species appear to be associated with evolution of extreme skull shapes [62]. Therefore, the evolvability of ontogenetic allometry may remain an important aspect of skull diversification in echolocating bats and warrants an in-depth comparative study.

### Shifts in intrinsic factors impacted evolutionary lability of the bat skull

Bats show a pattern of mosaic evolution [63] across the skull; different parts of the cranium and mandible diversified at different rates. Shifts away from the ancestral state of oral emission—to nasal emission or loss of echolocation—were associated with both changes in modularity or allometry, and rates of evolution across the skull. The loss of echolocation led to a slowing of cranial shape evolution, with the highest rates (albeit still slow compared to echolocating bats) restricted to the rostrum, the area with the most size-linked variability. It has been suggested that increases in modularity should be linked to increased morphological diversification, as different modules should be more “free” to evolve independently, and may more rapidly adapt to changing selective pressures [12, 31–33, 64]. Comparatively, more integrated structures may facilitate diversification by permitting rapid coordinated changes in shape across a suite of traits [14, 33, 65]. Consistent with the latter, our results show a shift in modularity in nasal-emitting bats towards a more integrated cranium (i.e., fewer modules) occurred concomitantly with increased rates of morphological evolution.

Increased morphological integration has been associated with increased rates of morphological evolution but lower disparity in the crania of other vertebrates [31]. However, nasal and oral emitting bats show similar levels of cranial disparity [35]. Nasal emission in bats is associated with a variety of both hard and soft tissue adaptations, including bony nasal domes, frontal concavities, “floating” premaxillae [37], modified turbinals [66], and elaborate nose leaves [67]. Consistent with the location of these major features, the rostrum is one of the fastest evolving regions in the cranium of nasal emitters. This structure also showed generalized increases in evolutionary rates (Fig. 4) and the lowest overall rate heterogeneity (see Table S5–6), supporting a broad adaptive process promoting rapid diversification of the cranium in these bats. This further highlights multiple pathways that have allowed bats to accomplish the same sensory mode in spite of increased cranial integration [5, 68].

One of the major transitions to nasal emission occurred within the Phyllostomidae, a long-supposed adaptive radiation [69]. While phyllostomid skull shapes varied greatly in the context of a broadly sampled bat cranial morphospace, much of their disparity was distributed across a narrow band of morphospace [35]. It is possible the shift in intrinsic factors determining skull shape variation (i.e., integration) pre-disposed phyllostomids to more readily evolve along an axis of broad skull elongation/shortening in the light of new ecological opportunities in the Americas.

## Conclusions

We found considerable shifts in the intrinsic factors driving skull morphological variation as a response to sensory adaptations in bats. Even within echolocators, different modes of sound emission posed significantly different pressures on modularity and allometry. Loss of echolocation and reliance on vision appears to have renewed constraints seen in other mammals, and likely contributed to the low skull shape diversity of pteropodid bats. By comparison, the transition from oral to nasal emission was associated with higher skull shape diversification, despite decreases in cranial modularity. These early changes in the inherent ways morphological variation is structured may have biased further pathways for trait diversification in ecologically diverse groups. Bat skull evolution can help us better understand how intrinsic and extrinsic factors jointly produce extraordinary patterns of morphological disparity and associated functional and ecological diversity. These results also contribute to a growing discussion and reframing of intrinsic evolutionary “constraints”. Both allometry and integration have been hypothesized to limit shape variation, but each may enhance diversification rates overall. Indeed, increased cranial integration was associated with highly disparate skull shapes among clades like the Phyllostomidae. It also begs the question, how constraining are these “constraints” if they are evolutionarily labile, as observed across the different modes of echolocation in bats? Further study is needed to assess the extent to which these intrinsic factors limit or enhance trait evolution in the light of trait adaptation, and their evolvability across a broader taxonomic sampling than addressed here.

## Methods

### Data collection

To assess allometry, morphological modularity, and evolutionary rate heterogeneity across the bat skull (cranium and mandible), we used a 3D geometric morphometric dataset from our previous analysis of bat skull shape [35]. Briefly, we collected 36 landmarks and 5 curves represented by equidistant semi-landmarks from cranial models and 20 landmarks and 4 curves from the mandible models generated from μCT scans of 1–8 specimens per species [35] (Additional file 1: Description of Landmarks, Fig, S1). Missing data (e.g., landmarks absent on one side of the skull due to specimen damage) were first estimated by exploiting bilateral symmetry via reflected relabeling [70], and unpaired or bilaterally missing landmarks were estimated using Bayesian Principal Component Analysis [71–73]. Specimens were scaled to centroid size, transposed, and optimally rotated using generalized Procrustes superimposition, which removed the impact of scale and position from our shape dataset. Landmarks were subsequently averaged across species and across bilateral pairs after mirroring (i.e., only one side of the skull was analyzed after superimposition, and shape variation associated with asymmetry was not included in our analyses).

We divided the 202 species of bats represented in our dataset into three echolocation categories based on presence/absence of laryngeal echolocation (non-echolocators *n* = 28) and the primary mode of echolocation call emission (oral emitters *n* = 88; nasal emitters *n* = 86). We note that, while most pteropodid species are visually oriented non-echolocators, at least one genus (*Rousettus*) has independently evolved echolocation via tongue clicks [61, 74] and another genus (*Eonycteris*) has independently evolved echolocation via wing clicks [75]. As these echolocation modes are rare within bats and differ in functional and anatomical features from laryngeal echolocation, we grouped *Rousettus* and *Eonycteris* with other non-laryngeal echolocators. For all phylogenetically informed analyses, we used a subtree of all 202 species from a recent molecular phylogenetic analysis of Chiroptera [76]. For analyses within each of the three echolocation groups (oral-, nasal, and non-echolocators), we pruned this tree to species only represented by each group.

### Tests of evolutionary allometry

To test for allometric trends in bat skull shape, we determined the relationship between overall (centroid) size and shape of the cranium and mandible, respectively, using the R function “procD.allometry” from the package “geomorph”. We included echolocation mode (2 groups—laryngeal echolocators vs. non-echolocators, and 3 groups—non-echolocators, nasal emitters, and oral emitters) as a covariate, and used the test for homogeneity of slopes to determine whether allometric patterns differed across evolutionary shifts in echolocation mode. We then examined the relationship between skull size and shape after accounting for evolutionary relatedness, using a phylogenetic least squares regression as implemented in “procD.pgls” from the R package “geomorph”. Based on differences in size-shape relationships between echolocators vs. non-echolocators, and the low shape variation explained by size after phylogenetic correction (see “Results”), we used the original Procrustes superimposed coordinate data for all subsequent analyses across bats.

### Detection of morphological modules

We used both exploratory and confirmatory approaches to examine morphological modularity in the bat skull [77], and followed the methods detailed by Goswami [42], among others, that employ cluster analysis of inter-landmark variation to detect morphological modules. We calculated the covariance matrix of all pairwise landmark comparisons as the dot product of the landmark coordinates minus the mean configuration, and divided by the pooled variance of the two landmarks [42, 78]. While many previous analyses have examined intra-specific morphological modularity, we focus on the modularity of the bat skull across their entire radiation. As trait covariation may be strongly impacted by evolutionary relationships, we modified the calculation of the matrix of congruence coefficients following the approaches of Adams and Felice [54] based on the evolutionary rate matrix R (Eq. 1, C provides the phylogenetic covariance matrix). The R matrix is comprised of the diagonal elements, which express evolutionary trait variances (evolutionary rates), and the off-diagonals that express pairwise evolutionary trait covariances. To calculate the phylogenetically corrected matrix of correlation coefficients, we expressed R as a partitioned matrix of the 3D coordinates for each of two landmarks, L1 and L2 [24, 54] (Eq. 2). The individual coefficients (*r*) were calculated from matrix R as shown in Eq. 3 (and see Additional file 2 for R script). The trace of R_L1,L2_ gives the summed covariances across landmark coordinates, which is equivalent to the vector dot product of landmark coordinates as given by Goswami 2006, but including phylogenetic correction. The trace of R_L1,L1_ and R_L2,L2_ gives the summed coordinate variances per landmark.

1 $$ R=\frac{{\left(X-E(X)\right)}^t{C}^{-1}\left(X-E(X)\right)}{N-1} $$

2 $$ R=\left[\begin{array}{cc}{R}_{L1,L1}& {R}_{L1,L2}\\ {}{R}_{L2,L1}& {R}_{L2,L2}\end{array}\right] $$

3 $$ {r}_{L1,L2}=\frac{\mathrm{trace}\left({R}_{L1,L2}\right)}{\sqrt{\mathrm{trace}\left({R}_{L1,L1}\right)\ast \mathrm{trace}\left({R}_{L2,L2}\right)}} $$

We used eigen analysis and hierarchical cluster analysis to detect evolutionarily corrected morphological modules from the correlation matrix. We applied eigen analysis (using the R function “eigen”) to the correlation matrix; the number of axes with eigenvalues greater than would be expected by chance (based on a broken stick distribution) represented the best fit number of modules [79, 80]. For the cluster analysis, we converted correlation coefficients to Euclidean distances and clustered them using Ward’s method [42, 78, 79].

To estimate modules that reflected biologically realistic units (e.g., anatomically contiguous structures), we carried out a cluster analysis using the R package “ClustGeo,” which weights trait comparisons by a constraint matrix (e.g., geographic distances, adjacency matrices). We calculated D1 (the constraint matrix) as the pairwise Euclidean distances between landmarks in the mean landmark configuration, using a weight value selected using “choicealpha.” For consistency, we selected a single *α* value that minimized the loss of inertia for the two matrices (congruence and Euclidean distances of landmarks) across all datasets (*α* = 0.5). However, we found that small changes of *α* (± 0.1) did not substantially impact the modules detected in any of the datasets (results not shown). For each echolocation mode (non-echolocators, nasal-emitting laryngeal echolocators, oral-emitting laryngeal echolocators), we repeated the above analysis using the landmark coordinates and subtree for each group. We contrasted modules detected across species grouped by echolocation mode with modules detected across all bats using “cophylogenetic” plots (Additional file 1: Fig. S7–8), which rotate nodes in cladograms/phylogenies to optimize tip matching.

Across all bats and within each echolocation mode group, we evaluated the support for each of the detected sets of modules of the cranium and mandible using both simulation and likelihood approaches. We first tested the significance of each module set using the covariance ratio with phylogenetic correction [24]. We also examined whether each echolocation group has experienced a shift away from “typical” bat modularity (i.e., that observed across all bats), by testing for the significance of the “all bat” modules within each echolocator group. Then, to further test for changes in skull modularity across echolocation modes, we implemented the likelihood approach “EMMLi” [77], which allows to fit models that possess the same or different inter- and intra-module landmark covariation. As this approach can compare models of substantially different complexity and structure, we also considered a set of previously inferred models for bats, mammals, and vertebrates (Additional file 1: Table S1–2). These comprised two previously hypothesized sets of modules for the cranium, a vertebrate face-braincase two module system [52], and a mammalian six-module system [42], as well as a set of morphological modules developed for analyses of the phyllostomid mandible [43]. We applied EMMLi to the evolutionarily corrected landmark correlation matrix described above.

### Evolutionary rate variation across the bat skull

Lastly, we tested whether shifts in echolocation mode caused changes in rates of evolution across different regions of the skull [9, 81]. We follow the philosophical approach of two recent studies [77, 78] that treat each multidimensional landmark as the relevant unit of anatomical information, in contrast with approaches that integrate analyses across the coordinates of multiple landmarks simultaneously [9]. While this is in some cases more computationally efficient, it treats the individual coordinates (*x*, *y*, or *z*) as independent traits, capable of separately covarying with coordinates from other landmarks [78]. We examined variation in evolutionary rates across the bat skull using distance-based (Q-mode [46]) and likelihood-based (R-mode) approaches. The Q-mode approach contrasts evolutionary rates across multidimensional traits by calculating the Brownian Motion (BM) evolutionary rate (*σ*^2^) as a single parameter across multiple traits or multiple dimensions of a trait. Variation in evolutionary rates across structures is determined by contrasting the ratio of evolutionary rates between two modules (or for more than two modules, the fastest and slowest rates) to those observed in character sets produced by simulating BM evolution under a constant rate across all traits/landmarks [9]. We tested whether evolutionary rates varied across morphological modules as well as across individual landmarks, with each landmark representing a module with 3 dimensions, using “compare.multi.rates.geomorph” from the R package “geomorph.”

The above approach is limited in comparing models of varying complexity. Therefore, we reformulated the calculation of the R matrix for macroevolutionary modeling to permit multiple dimensions per trait. We derived this R matrix (Eq. 4) using the multivariate independent contrast defined by McPeek et al. [82], which expresses the total evolutionary change per landmark. Like the formulation of the matrix used for module detection above, the elements of the R matrix are equivalent to the sum of the evolutionarily transformed trait variances and covariances across the dimensions of each landmark. Equation 5 gives the reformulated likelihood for a BM model using the adjusted R matrix. In Eq. 5, the vectors X, Y, and Z supply the coordinates of each landmark across all species, matrix C is the phylogenetic variance-covariance matrix derived from the phylogeny, and vectors E(X)/E(Y)/E(Z) provide the expected values of each trait under a BM process. The diagonal elements of the R matrix provide the multivariate evolutionary rate of each three-dimensional landmark, while the off-diagonal elements provide the inter-landmark covariations, again across all three dimensions simultaneously. Additional file 1 provides the full derivation for evolutionary rates calculated by independent contrasts or PGLS methods.

4 $$ {R}_{L\left(X,Y,Z\right)}=\frac{1}{N}\left({\left(X-E(X)\right)}^t{(C)}^{-1}\left(X-E(X)\right)+{\left(Y-E(Y)\right)}^t{(C)}^{-1}\left(Y-E(Y)\right)+{\left(Z-E(Z)\right)}^t{(C)}^{-1}\left(Z-E(Z)\right)\right) $$

5 $$ Log(L)=-\frac{1}{2}\left({\left(X-E(X)\right)}^t{\left(R\bigotimes C\right)}^{-1}\left(X-E(X)\right)+{\left(Y-E(Y)\right)}^t{\left(R\bigotimes C\right)}^{-1}\left(Y-E(Y)\right)+{\left(Z-E(Z)\right)}^t{\left(R\bigotimes C\right)}^{-1}\left(Z-E(Z)\right)+\log \left|R\bigotimes C\right|+ Nlog\left(2\pi \right)\right) $$

We calculated the likelihood and Akaike information criterion scores of the full evolutionary rate matrix (all rates vary), R matrices constraining the diagonal to the average rate value (one rate), and different rates per modules (three to seven rates). We visualized variation in evolutionary rates using functions “interp” and “image.plot” from the packages “akima” and “fields”, respectively [83, 84]. We linearly interpolated evolutionary rates across the 2D lateral and dorsal views of the cranium and mandible using the consensus configurations of landmark coordinates. We used these only to visualize areas of high or low rates, and these plots are not meant to represent rate estimates at any given point on the skull. Regions with lower sampling of landmark coordinates are obviously more poorly informed by this approach.

## Supplementary Information

**Additional file 1.** Arbour et al. supp mat.docx - supplementary equations and results, including figures and tables denoted by S.

**Additional file 2.** R script for phylogenetic congruence matrix.

**Additional file 3.** R script for evolutionary rate model fitting.

## Data Availability

The phylogenetic tree used in this study is available in the supplementary material of Shi and Rabosky (2015) at 10.1111/evo.12681. All landmark data is available through the supplementary materials of Arbour et al. (2019) at https://www.nature.com/articles/s41467-019-09951-y.
