# Supplementary material for: Sensory adaptations reshaped intrinsic factors underlying morphological diversification in bats
Source: BMC Biol. 2021 Apr 30;19:88. doi: 10.1186/s12915-021-01022-3 (PMC8086122; doi:10.1186/s12915-021-01022-3)
Supplement: Supplementary file 1 — Additional file 1. Arbour et al. supp mat.docx - supplementary equations and results, including figures and tables denoted by S. [file 12915_2021_1022_MOESM1_ESM.docx]

Sensory adaptations reshaped intrinsic factors underlying morphological diversification in bats

J.H. Arbour, A.A. Curtis, S.E. Santana

**SUPPLEMENTARY MATERIALS**

Description of Landmarks

**Mandible Landmarks**

1. Anteriormost point on the mandibular symphysis

2-3. Anteriormost point on canine alveolus

4-5. Anteriormost point on premolar alveolus

6-7. Anteriormost point of first molar alveolus

8-9. Lateralmost point on mandibular condyle

10-11. Medialmost point on mandibular condyle

12-13. Inflection point on the posterior profile between the mandibular condyle and the angular process

14. Ventralmost point on the mandibular symphysis

15-16. Posteriormost point on the angular process

17-18. Posteriormost point on the last molar alveolus

19-20. Anterior inflection point on the articular surface, midpoint between L8/9 and L10/11.

*Curves with equidistant sliding semi-landmarks*

1-2, From L17/18 to L19/20, along the dorsal profile of the coronoid process. (blue)

3-4, From L14 to L15-16, along the ventral profile of the ramus and angular process. (red)

**Cranial Landmarks**

1-2. Anteriormost point of premaxilla

3-4. Anteriormost point on canine alveolus

5-6. Anteriormost point on premolar alveolus

7-8. Anteriomost point on the first molar alveolus

9-10. Posteriormost point on the last molar alveolus

11-12. Ventralmost point on pterygoid hamulus

13-14. Medialmost margin of the mandibular fossa

15-16. Ventralmost point on mastoid process

17-18. Dorsalmost point on the external edge of the auditory meatus

19-20. Ventralmost point on the external edge of the auditory meatus

21-22. Lateralmost point on occipital condyle

23. Dorsal border of foramen magnum

24. Ventral border of foramen magnum

25. Posteriormost point on midline of palate

26. Anteriormost point on midline of the complete palate

27. Anteriormost point on the midline of the nasals

28 Posteriormost point on the intersection of the lambdoidal and sagittal crests

29-30. Anteriormost point on the inflection of the orbit

31-32. Posteriormost point on the intersection of the zygomatic arch and braincase

33-34. Ventralmost point of the insertion of the zygomatic arch on the maxilla

35-36 Lateralmost point on the margin of the mandibular fossa

*Curves with equidistance sliding semi-landmarks:*

1. From L27 to L28, along the dorsal midline of the cranium (blue)

2-3. From L29/30 to L31/32, along the dorsal profile of the zygomatic arch (red)

4-5. From L33/34 to L35/36, along the ventral profile of the zygomatic arch (yellow)


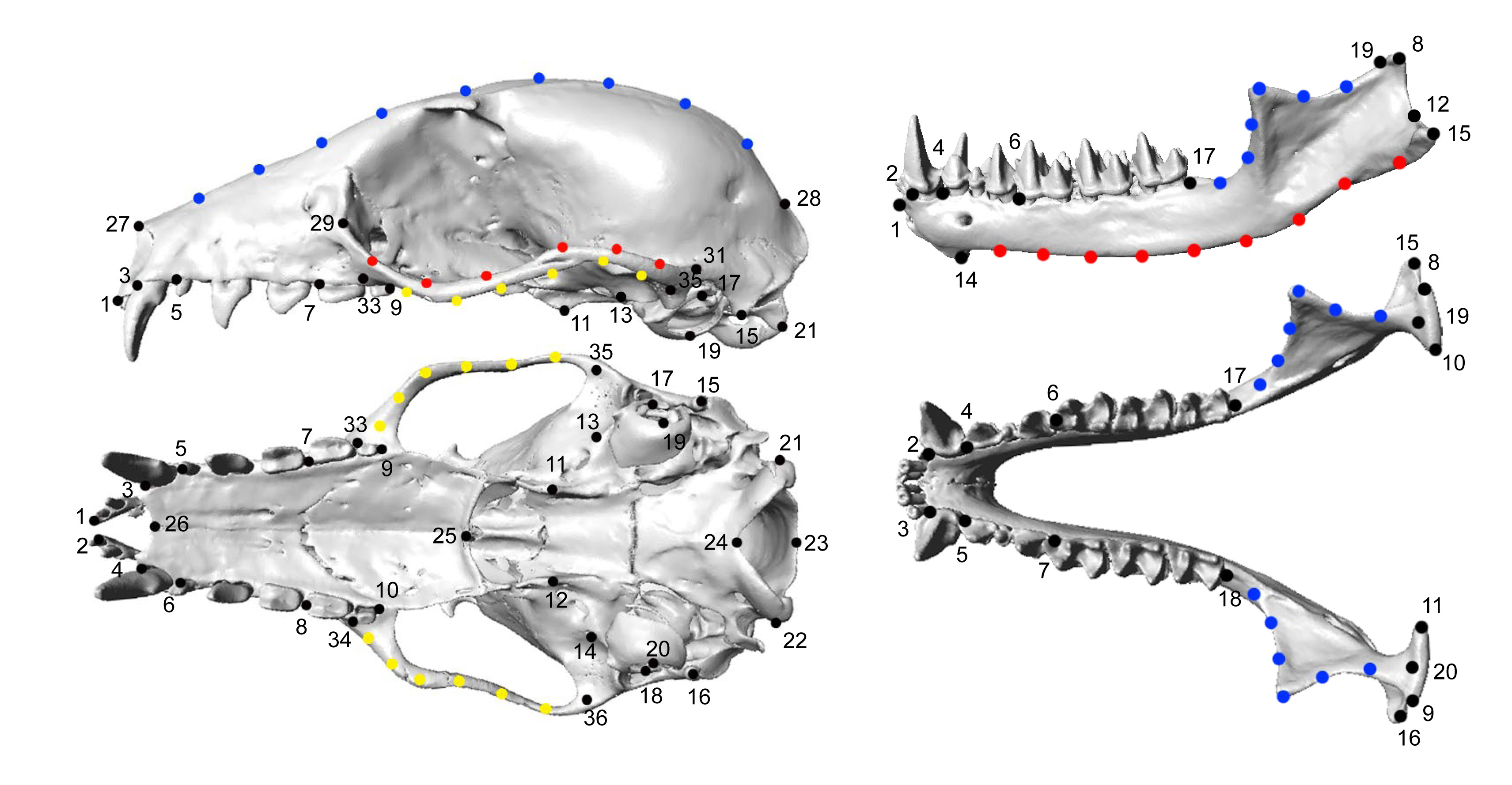


Fig. S1: Location of 3D geometric morphometric landmarks on the cranium and mandible of *Eonycteris spelaea*. Landmarks are given by black points, semi-landmarks by coloured points (see landmark list above). Reproduced from: Arbour et al. 2019. Signatures of echolocation and dietary ecology in the adaptive evolution of skull shape in bats. *Nature Communications* 10: 2036 [35]

Table S1: Alternative hypotheses of modularity in the bat cranium (ant indicates the anterior end of the line, pos indicates the posterior end). See above description of landmarks for full details.

| Landmark | Face (f) /Braincase (b) | Goswami 6 mammal modules [41] |
| --- | --- | --- |
| premaxilla | f | oral-nasal |
| canine | f | oral-nasal |
| premolar | f | oral-nasal |
| molar anterior | f | molar |
| molar posterior | f | molar |
| pterygoid hamulus | b | zygomatic-pterygoid |
| mandibular fossa | b | zygomatic-pterygoid |
| mastoid | b | basicranium |
| dorsal auditory meatus | b | basicranium |
| ventral auditory meatus | b | basicranium |
| lateral occiptal condyle | b | basicranium |
| dorsal zygomaticomatic 1 orbit | f | orbit |
| dorsal zygomatic 2 (ant) | f | orbit |
| dorsal zygomatic 3 (ant) | f | zygomatic-pteryoid |
| dorsal zygomatic 4 (ant) | f | zygomatic-pterygoid |
| dorsal zygomatic 5 (pos) | f | zygomatic-pterygoid |
| dorsal zygomatic 6 (pos) | f | zygomatic-pterygoid |
| dorsal zygomatic 7 (pos) | f | zygomatic-pterygoid |
| dorsal zygomatic 8 (pos) | b | zygomatic-pterygoid |
| dorsal zygomatic 1 (insert) | f | molar |
| dorsal zygomatic 2 (ant) | f | molar |
| dorsal zygomatic 3 (ant) | f | zygomatic-pterygoid |
| dorsal zygomatic 4 (ant) | f | zygomatic-pterygoid |
| dorsal zygomatic 5 (pos) | f | zygomatic-pterygoid |
| dorsal zygomatic 6 (pos) | f | zygomatic-pterygoid |
| dorsal zygomatic 7 (pos) | f | zygomatic-pterygoid |
| dorsal zygomatic 8 (fossa) | b | zygomatic-pterygoid |
| foramen magnum (pos) | b | basicranium |
| foramen magnum (ant) | b | basicranium |
| palate (pos) | f | molar |
| palate (ant) | f | oral-nasal |
| dorsal midline 1 nasals | f | oral-nasal |
| dorsal midline 2 (ant) | f | oral-nasal |
| dorsal midline 3 (ant) | f | orbit |
| dorsal midline 4 (ant) | b | orbit |
| dorsal midline 5 (ant) | b | vault |
| dorsal midline 6 (ant) | b | vault |
| dorsal midline 7 (pos) | b | vault |
| dorsal midline 8 (pos) | b | vault |
| dorsal midline 9 (pos) | b | vault |
| dorsal midline 10 (pos) | b | vault |
| dorsal midline 11 (crests) | b | vault |

Table S2: Alternative hypotheses of modularity in the bat mandible. (ant indicates the anterior end of the line, pos indicates the posterior end). See above description of landmarks for full details.

| Description | Monteiro and Nogueira 5 [44] |
| --- | --- |
| canine | anterior alveolar |
| premolar | anterior alveolar |
| first molar | posterior alveolar |
| lateral condyle | condyle |
| medial condyle | condyle |
| inflection on ramus | condyle |
| last molar | posterior alveolar |
| coronoid 1 (ant) | coronoid |
| coronoid 2 (ant) | coronoid |
| coronoid 3 (ant) | coronoid |
| coronoid 4 (pos) | coronoid |
| coronoid 5 (pos) | coronoid |
| coronoid 6 (pos) | coronoid |
| dorsal condyle | condyle |
| ventral 1 (ang) | angular |
| ventral 2 (ang) | angular |
| ventral 3 (ang) | angular |
| ventral 4 (body-pos) | posterior alveolar |
| ventral 5 (body-pos) | posterior alveolar |
| ventral 6 (body-pos) | posterior alveolar |
| ventral 7 (body-pos) | posterior alveolar |
| ventral 8 (body-ant) | posterior alveolar |
| ventral 9 (body-ant) | posterior alveolar |
| ventral 10 (body-ant) | posterior alveolar |
| symphysis (anterior) | anterior alveolar |
| ventral 11 (symphysis) | anterior alveolar |

Derivation of Evolutionary Rates Per Landmarks

The Brownian Motion evolutionary rate parameter may be expressed both from independent contrasts and PGLS (phylogenetic generalized least squares) approaches. McPeek [82] calculated the value of independent contrasts for multivariate traits as described in equation 1. For orthogonal and similarly scaled traits (e.g., geometric morphometric landmark coordinates), this contrast can be equivalently expressed as the square-root sum of squares of the contrasts in each dimension (shown for 2D and 3D coordinates below, Eq. 2 and 3 respectively). The BM evolutionary rate parameter (σ2) can be calculated as the average squared independent contrast for a trait y and a group of N taxa (Eq. 4). For orthogonal trait axes, the BM rate parameter can therefore also be expressed as the average sum of squared contrasts for each of 2 (Eq. 5) or 3 (Eq. 6) dimensions.

Eq. 1 $c_{X|1,2}= \frac{X_{1}+X_{2}}{\sqrt{v_{1}+v_{2}}}$

Eq. 2 $c_{xy} \sqrt{c_{x}^{2}+c_{y}^{2}}$

Eq. 3 $c_{xyz}=\sqrt{c_{x}^{2}+c_{y}^{2}+c_{z}^{2}}$

Eq. 4 $\sigma^{2}=\frac{1}{N-1} \sum_{i}^{N-1} c_{i}^{2}$

Eq. 5 $\sigma_{xy}^{2}=\frac{1}{N-1} \sum_{i}^{N-1} \left( c_{xi}^{2}+c_{yi}^{2} \right)$

Eq. 6 $\sigma_{xyz}^{2}=\frac{1}{N-1} \sum_{i}^{N-1} \left( c_{xi}^{2}+c_{yi}^{2}+c_{zi}^{2} \right)$

The PGLS expression of the one-dimensional BM rate parameter is as shown in Eq. 6. It is important to note that this calculation produces the biased BM rate (divided by N) rather than the unbiased evolutionary rate (divided by N-1). However, this is typical of many evolutionary model fitting approaches for continuous traits implemented in R software packages and the previous literature on BM model likelihoods [45, 50]. When the same denominator is used (N or N-1), Eq. 4 vs. 7 and Eq. 6 vs. 8 produce equivalent rates under BM.

Eq. 7. $\sigma^{2}=\frac{{(y-E\left( y \right))}^{t}C^{-1}(y-E\left( y \right))}{N}$ $E\left( y \right)=\left( 1^{t}C^{-1}1 \right)^{-1}(1^{t}C^{-1}y)$

Eq. 8. ${\sigma_{xyz}}^{2}=\frac{1}{N}\left[ \left( x-E\left( x \right) \right)^{t}C^{-1}\left( x-E\left( x \right) \right)+\left( y-E\left( y \right) \right)^{t}C^{-1}\left( y-E\left( y \right) \right)+\left( z-E\left( z \right) \right)^{t}C^{-1}\left( z-E\left( z \right) \right) \right]= \frac{1}{N} \sum_{i} c_{i,x}^{2}+c_{i,y}^{2}+c_{i,z}^{2}$

Eq. 9 $Log\left( L \right)=-\frac{1}{2}\left( {(x-E\left( x \right))}^{t}\left( \sigma^{2}C \right)^{-1}\left( x-E\left( x \right) \right)+\left( y-E\left( y \right) \right)^{t}\left( \sigma^{2}C \right)^{-1}\left( y-E\left( y \right) \right)+\left( z-E\left( z \right) \right)^{t}\left( \sigma^{2}C \right)^{-1}\left( z-E\left( z \right) \right)+\log\left| \delta^{2}C \right|+Nlog\left( 2\pi\right) \right)$

The multivariate implementation of this evolutionary rate equation allows for the simultaneous calculation of the evolutionary rate of multiple multi-dimension at the same time, assuming all traits have the same dimensionality (e.g., like geometric morphometric landmarks). For univariate characters, the R matrix can be calculated as follows in Eq. 10. Its diagonal elements are the individual univariate evolutionary rates (Eq. 4 and 7), while the off-diagonal elements give the covariances between traits [50].

Eq. 10 $R_{Y}=\frac{1}{N}\left( Y-E\left( Y \right) \right)^{t}\left( C \right)^{-1}\left( Y-E\left( Y \right) \right)=\left[ \begin{matrix} \sigma_{1}^{2} & {cov}_{1,2} \\ {cov}_{1,2} & \sigma_{2}^{2} \end{matrix} \right]$

Eq. 11 $R_{L(X,Y,Z)}=\frac{1}{N}\left( \left( X-E\left( X \right) \right)^{t}\left( C \right)^{-1}\left( X-E\left( X \right) \right)+\left( Y-E\left( Y \right) \right)^{t}\left( C \right)^{-1}\left( Y-E\left( Y \right) \right)+{(Z-E\left( Z \right))}^{t}{(C)}^{-1}(Z-E\left( Z \right)) \right)=\left[ \begin{matrix} \sigma_{L1}^{2} & {cov}_{L1,L2} \\ {cov}_{L1,L2} & \sigma_{L2}^{2} \end{matrix} \right]$

Eq. 12: $\mathrm{Log}\left( L \right)=-\frac{1}{2}\left( \left( X-E\left( X \right) \right)^{t}\left( R\bigotimes C \right)^{-1}\left( X-E\left( X \right) \right)+\left( Y-E\left( Y \right) \right)^{t}\left( R\bigotimes C \right)^{-1}\left( Y-E\left( Y \right) \right)+ \left( Z-E\left( Z \right) \right)^{t}\left( R\bigotimes C \right)^{-1}\left( Z-E\left( Z \right) \right)+ log \left| R\bigotimes C \right|+Nlog\left( 2\pi\right) \right)$

This rearrangement not only results in a smaller R matrix and fewer parameters for estimation under maximum likelihood approaches (prior implementations included each landmark dimension as its own trait, resulting in a R matrix diagonal of 3X length for our data), but also better allows for comparisons between multiple rate modules and models of varying numbers of parameters than similar Q-mode approaches (which only compare only two modules, or the difference between the fastest and slowest evolving modules, see “compare.multi.evol.rate” in the package “geomorph”).

As the number of free parameters in the matrix R increases exponentially with the number of traits a full maximum likelihood optimization of the evolutionary rate matrix is not currently computationally feasible. For a comparison between the Q-mode and R-mode methodologies, we resolve this difficulty using two approaches. Firstly, by setting trait covariances to zero to calculate the likelihood of the evolutionary rates under trait independence. This is similar to the approach used in the “surface” and “l1ou” methods for adaptive landmark model fitting across multiple axes.

The diagonal values of the rate matrix R in eq. 11 are equivalent to the σ^2^_mult_ parameter [45] (Fig. S2), as determined using the R function “compare.mutli.evol.rates” in the R package “geomorph” and assigning each landmark’s coordinates as a single module. It is important to note, that the rates calculated by this function are divided by the dimensions of each module, which is consistently 3 for our data, hence the 3:1 relationship between the calculated values. The rates are otherwise identical using this R mode and the previously published Q-mode approach, and the ratios of evolutionary rates are identical. Likelihood extensions of this R matrix have the benefit of allowing for the comparison of models with different numbers of modules while accounting for model complexity, in contrast with the Q-mode approach, which can only contrast two modules (greater numbers of modules compare the highest and lowest rates only in the geomorph implementation, while all other rates are ignored).


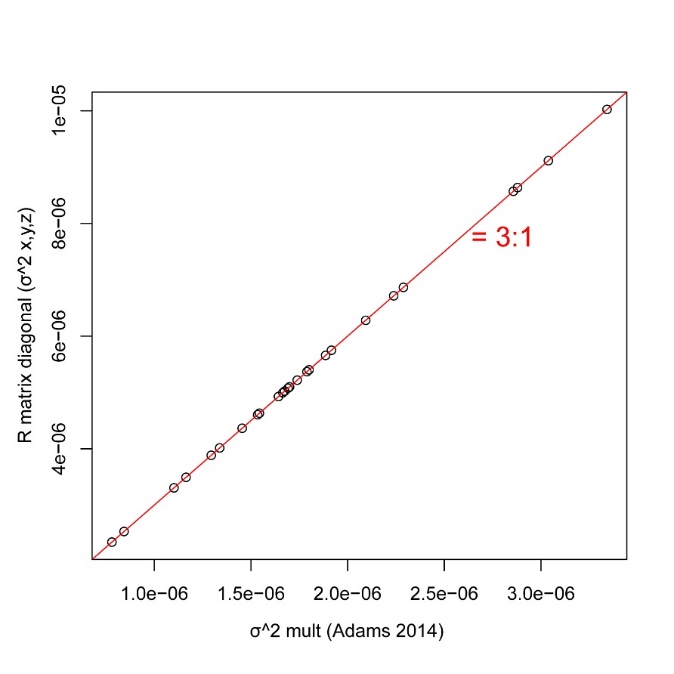


Fig. S2: Evolutionary rates calculated per landmark for the mandible dataset across all bats. Rates from the diagonal elements of the R matrix from equation 10, vs. sigma2mult calculated from the function “compare.multi.evol.rates” in the R package “geomorph”. The latter calculates the average rate across the dimensions of the module dimensions (here landmark, and therefore always 3). The red line shows a 3:1 relationship, which indicates mathematical equivalency.

We implemented an approach similar to that used in the “compare.multi.evol.rates” function by producing a constrained R matrix with trait covariances. In the aforementioned function, the diagonal elements of the observed R matrix (R.obs) are replaced by the global σ^2^_multi_ while the trait covariances are unchanged. The resulting matrix is not positive semidefinite, and “compare.multi.evol.rate” uses the function “nearPD” to produce the closest positive semi-definite matrix. However, we observed that the transformation as implemented in the geomorph function result in diagonal values of R differing from equality, and thus inflating the variation in simulated evolutionary rates (see Fig. S3). We have taken a different approach in constructing a constrained R matrix to calculate the likelihood of the singular or modularity rates based on our equation above. We convert the observed R matrix (an evolutionary trait variance-covariance matrix) to a correlation matrix by calculating the diagonal matrix D_1_ using the square root of the diagonal elements of the observed R matrix (R.obs) and transforming R.obs as follows.

$$R.cor= D_{1}^{-1}R.obs D_{1}^{-1}$$

We then rescale the phylogenetic correlation matrix (R.cor) based on the diagonal matrix D_2_, which includes the square root of the mean of the diagonal elements of the corresponding modules (or the mean of all rates for the single rate model).

$$R.constr= D_{2} R.cor D_{2}$$

This resulting constrained R matrix is positive semi-definite, incorporates trait covariance while maintaining the relative (standardized) relationships across all traits and does not produce variable rates under the one rate model (see Fig, S3). We emphasize that the likelihood of the constrained R matrices do not represent the full maximum likelihood solution for these models (as is the case with the “compare.evol.rates.multi” simulation-based, Q-mode approach). However, this is a limitation that impacts many multivariate macroevolutionary model approaches [10, 46] and has not been fully computationally resolved for datasets of the sizes generally experienced in highly multidimensional datasets like geometric morphometric landmark data. We hope that further implementations of this approach may be able to resolve the full maximum likelihood optimization of trait covariances, and acknowledge that the results herein will likely vary somewhat from full implementations of this approach.


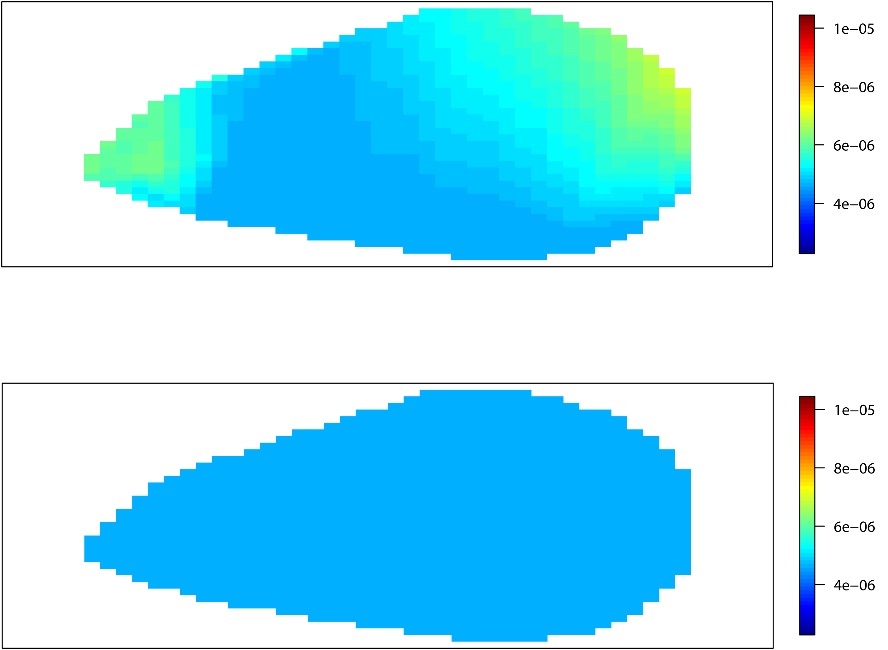


Fig. S3: Comparisons between evolutionary rates (σ^2^ = diag(R) ) produced using: top) nearPD approach as implemented in “compare.multi.rate.evol”, and bottom) the R rescaling approach described in the methods. The scale shows the full range of evolutionary rates observed across the full (n=202) dataset. The “compare.multi.rate.evol” approach produces rates that differ from equality and span a significant portion of the range of values in the observed dataset.

Statistical Performance of Multivariate Evolutionary Rates Model

We examined the statistical performance of the likelihood model of multivariate evolutionary rates described above using ΔAIC values across varying evolutionary rate ratios (the comparison between the highest and lowest evolutionary rates) and:

1) Varying numbers of species/taxa (25, 50, 100 and 150) – 4 landmarks

2) Varying numbers of landmarks (4, 8 and 12) – 50 species

3) Varying numbers of landmarks per module (out of eight landmarks, 1 vs. 7 landmarks, 2 vs. 6 landmarks, 3 vs. 5 landmarks and 4 vs. 4 landmarks (equivalent to the ratio used in the prior two options) – 50 species

4) Varying covariation among traits (standardized evolutionary correlations of 0, 0.25, 0.5 and 0.75). Across conditions 1-3 a value of 0.5 was used. Four landmarks and 50 species used.

For each of the above conditions, landmarks were assigned to one of two modules, in which the evolutionary rates were either 1 or a value between 1 and 4 (1, 1.25, 1.5, 1.75, 2, 2.5, 3, 4). This range overlapped with the observed rate ratios observed in our datasets using both the R and Q-mode approaches (Tables S5 and S6; note ratios above 4 always supported the generating model). Simulated landmark datasets were generated using the functions “pbtree” and “simchar” from the packages “phytools” and “Geiger” respectively [47, 49]. We contrasted the support for the generating model (2 modules of evolutionary rates) as the frequency of simulated datasets for which the ΔAIC of modular rates equalled zero (best fit model). We also determined the frequency at which a model of equal rates was poorly supported, as values of ΔAIC > 2, as this is often considered the minimum for support of the best fit model over others [48]. We compared model support using constrained R matrices that included or excluded trait covariation (regardless of the generating rate matrix).

We found that the likelihood approach to multivariate evolutionary rates performed well under conditions of rate heterogeneity and homogeneity. When generating rates were equal, the frequency of simulated character sets that supported rate heterogeneity (modular rates ΔAIC = 0) was ≤ 2.6% across all conditions examined, and equal rates models were poorly supported (ΔAIC > 2) across <1% of simulations. The correct model was supported across a majority of simulated (ΔAIC = 0 and ΔAIC of one rate model > 2) when the ratio between the evolutionary rates was greater than 1.5 to 1.75 across all conditions. Support for the generating model was increased with 1) greater numbers of species, 2) greater numbers of landmarks, 3) when landmarks were more evenly distributed across modules, and 4) when traits covaried.


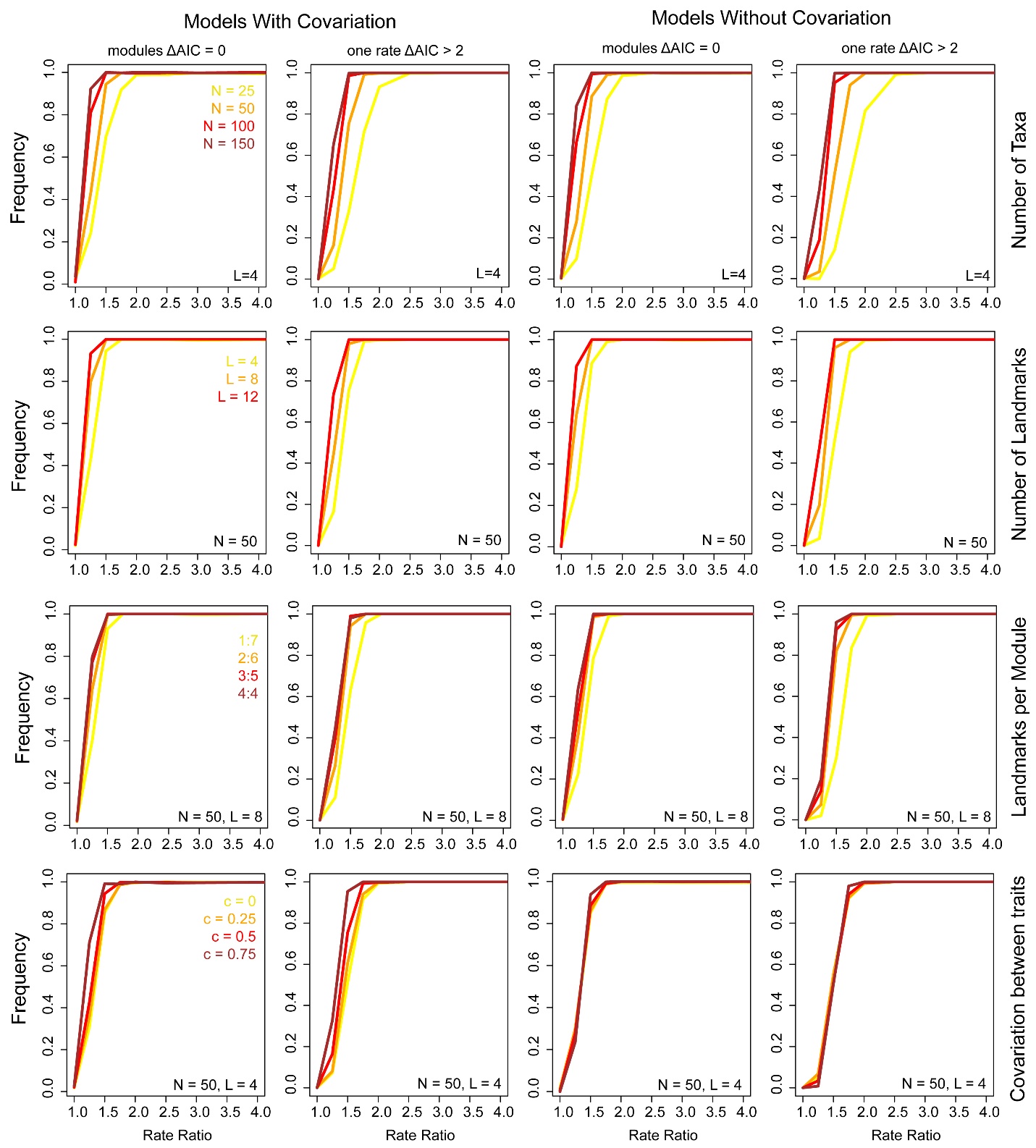


Fig. S4: Support for models of multivariate evolutionary rate heterogeneity across simulated datasets varying in the number of species, the numbers of landmarks, the number of landmarks per evolutionary module and the level of trait covariation. Graphs show the frequency of support for modular evolutionary rates, both when modular rates represent the best model (ΔAIC = 0) and when a model of rate homogeneity is poorly supported (ΔAIC > 2).

Lastly, we contrasted the fit of models supporting modular evolutionary rates and complete rate heterogeneity by comparing the support for these two models across a dataset in which all rates varied. We followed the procedure described using 50 species and 4 landmarks, in which the minimum evolutionary rate was 1, the highest rate was a value between 1 and 5 (1, 1.25, 1.5, 1.75, 2, 2.5, 3, 4, 5), and the two other rates were equally spaced between the minimum and maximum values. For example, when the maximum rate was 2, the other two rates equalled 1.33 and 1.67. We assigned each of these rates to the landmarks in two modules in differing combinations: R1 and R2 vs. R3 and R4, R1 and R3 vs. R2 and R4, R1 and R4 vs. R2 and R3.

When all evolutionary rates varied, but high and low rates were grouped together (R1+R2 vs. R3+R4), the modular rate model was supported over the all rates varying model when the ratio between the max and min rates ranged between 1.25 and 3. However, all other combinations of rates found strong support for the all rates vary model over both the modular rates and one rate models. As the number of modules increases in real datasets this would likely not be of great concern, as the chance of modules inadvertently grouping landmarks evolving under similar rates (but generated by different processes) by chance alone would decline. Such a condition may occur when a gradient of evolutionary rates occur across the length or span of a structure and low or high rates may occur proximate to one another, and so the spatial distribution of evolutionary rates should be examined in light of this.


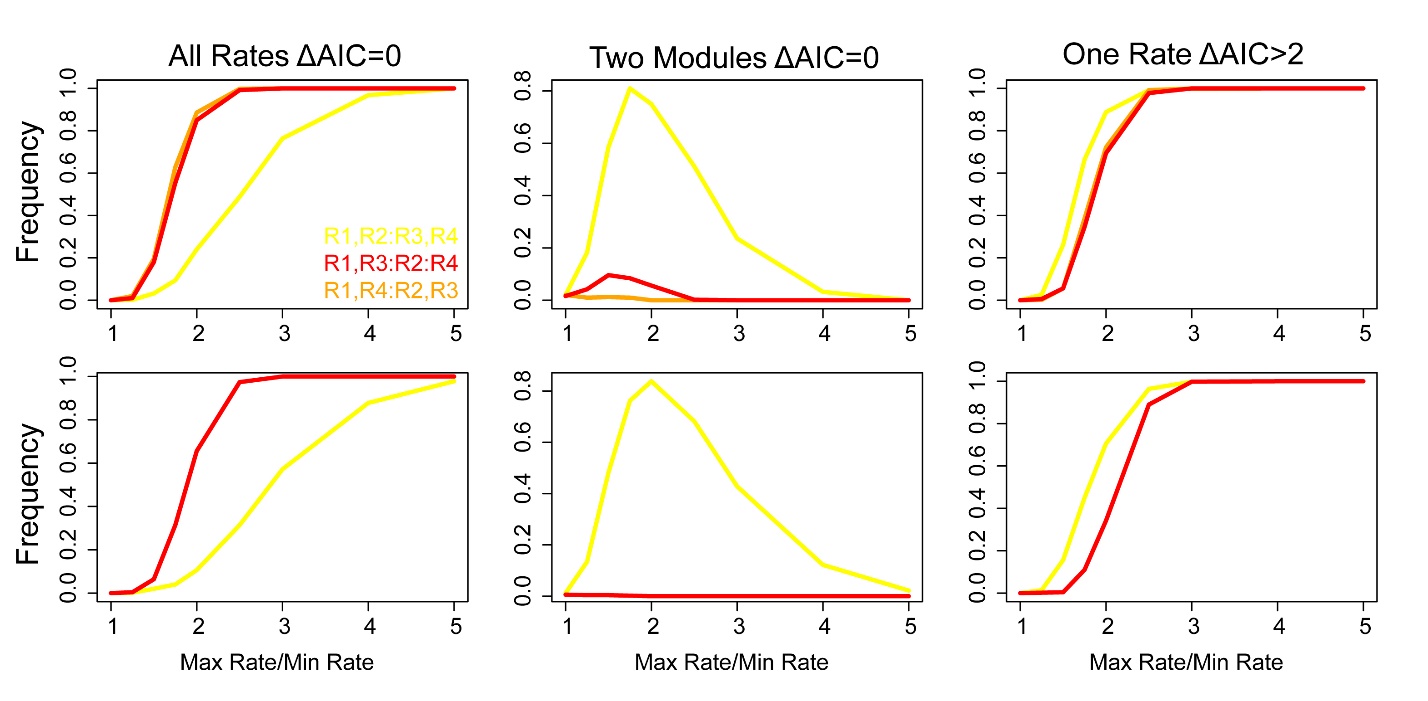


Fig. S5: Support for models of multivariate evolutionary rate heterogeneity across simulated datasets with four different evolutionary rates. Graphs show the frequency of support for modular evolutionary rates, when left) all rates varying represent the best model (ΔAIC = 0) middle) modular rates represent the best model (ΔAIC = 0) and left) when a model of rate homogeneity is poorly supported (ΔAIC > 2). Top row) models include trait covariation, bottom) models exclude trait covariation.

Evolutionary allometry within Echolocator Groups


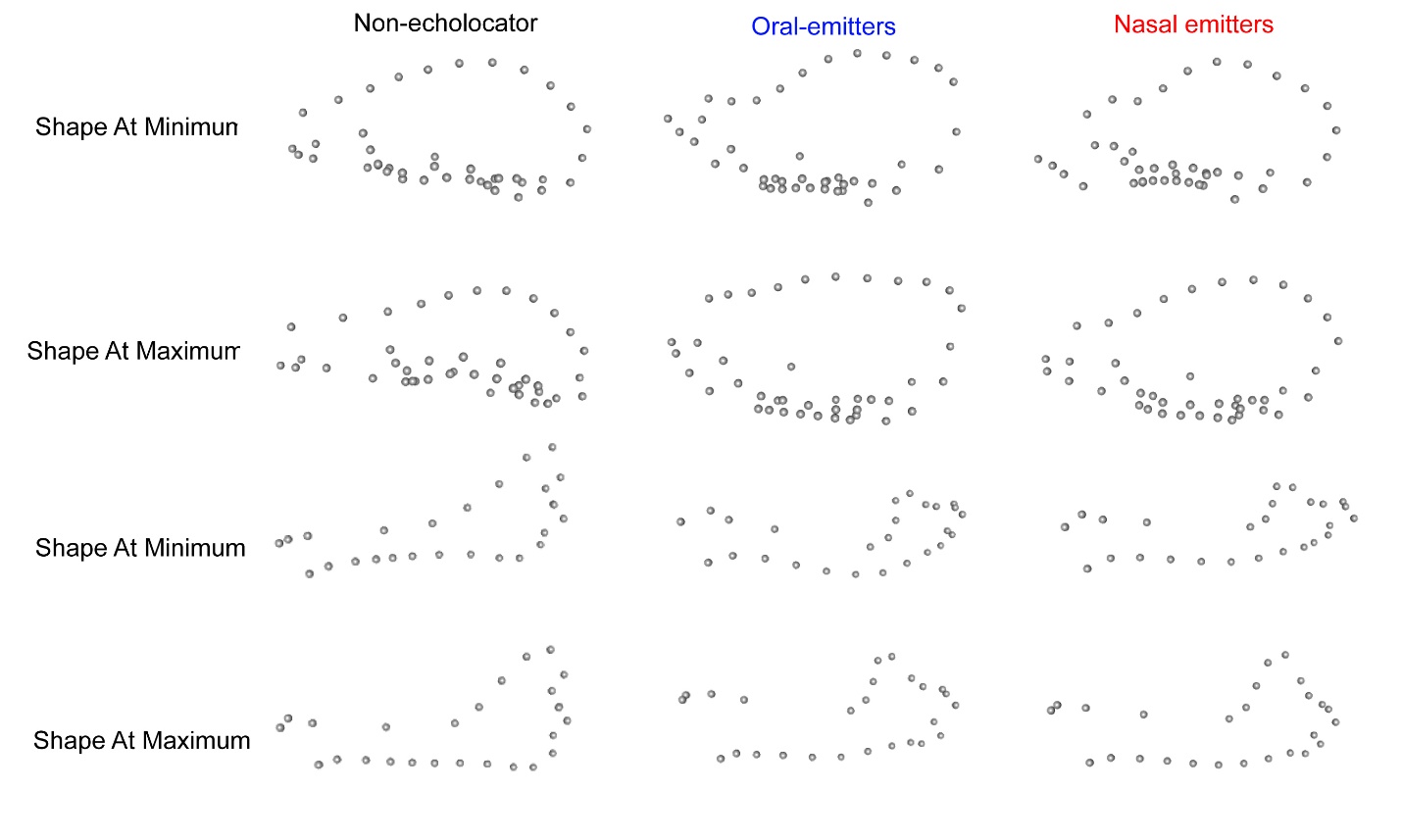


Fig. S6: Patterns of allometry in the cranium and mandible within echolocator groups. Shapes represent the landmark coordinate configurations calculated at the maximum and minimum centroid size of each of the datasets using the inferred allometric relationships.


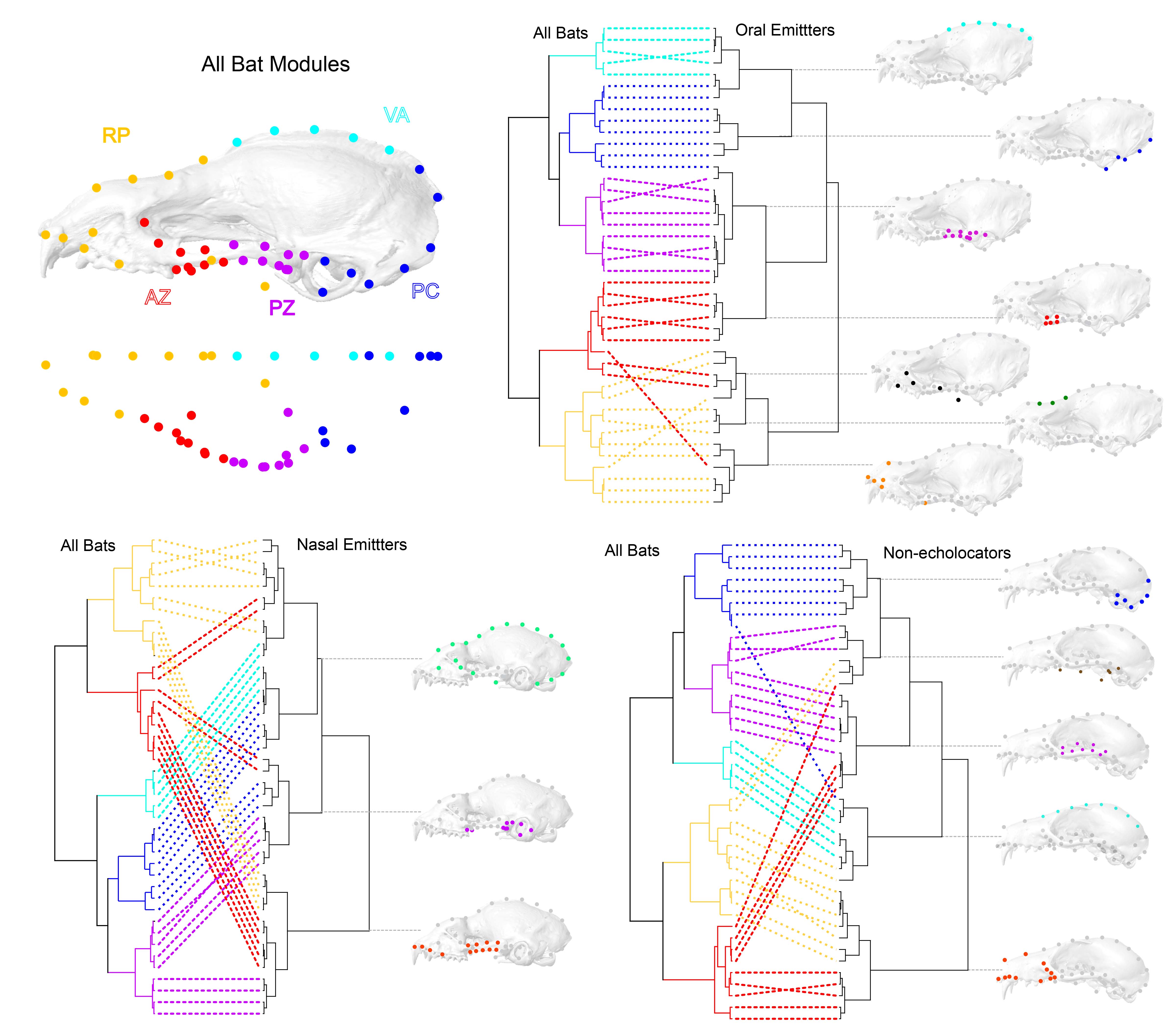


Fig. S7: Detection of bat cranial morphological modules based on cluster analysis of a phylogenetic congruence matrix. Modules detected across 202 bat species include: RP – rostrum/palate/pterygoid, VA – vault, AZ – anterior zygomatic arch, PZ – posterior zygomatic arch, PC – posterior cranium encompassing the basicranium, auditory bulla and the intersection of the lambdoidal and sagittal crests. Cladograms illustrate the relationship between landmarks within each echolocator group, and dashed connecting lines are colored by the module represented in the all bats dataset. For example – while the PZ landmarks most strongly covary with the PC landmarks in the all bats dataset, the PZ landmarks more strongly covary with AZ landmarks within oral echolocators. Example landmarks show the consensus configuration in lateral view within each dataset and representative crania and mandibles have been warped to match these configurations.


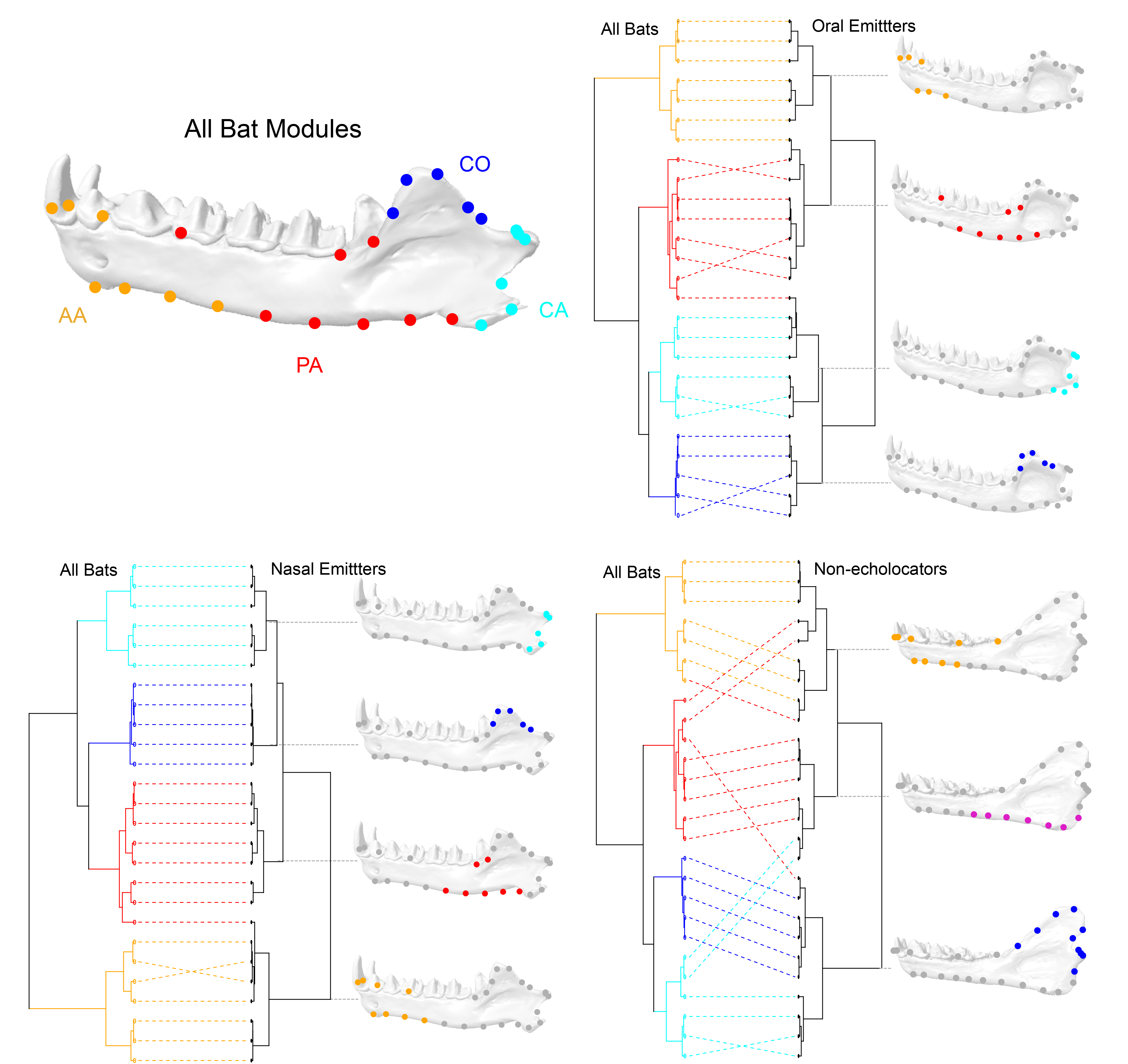


Fig. S8: Detection of bat mandible morphological modules based on cluster analysis of a phylogenetic congruence matrix. Modules detected across 191 bat species include: AA – anterior alveolus region, PA – posterior alveolus region, CO – coronoid process, CA – condyle and angular process. Cladograms illustrate the relationship between landmarks within each echolocator group, and dashed connecting lines are colored by the module represented in the all bats dataset. Example landmarks show the consensus configuration for the mandible in lateral view within each dataset and representative crania and mandibles have been warped to match these configurations.

Evolutionary Modularity


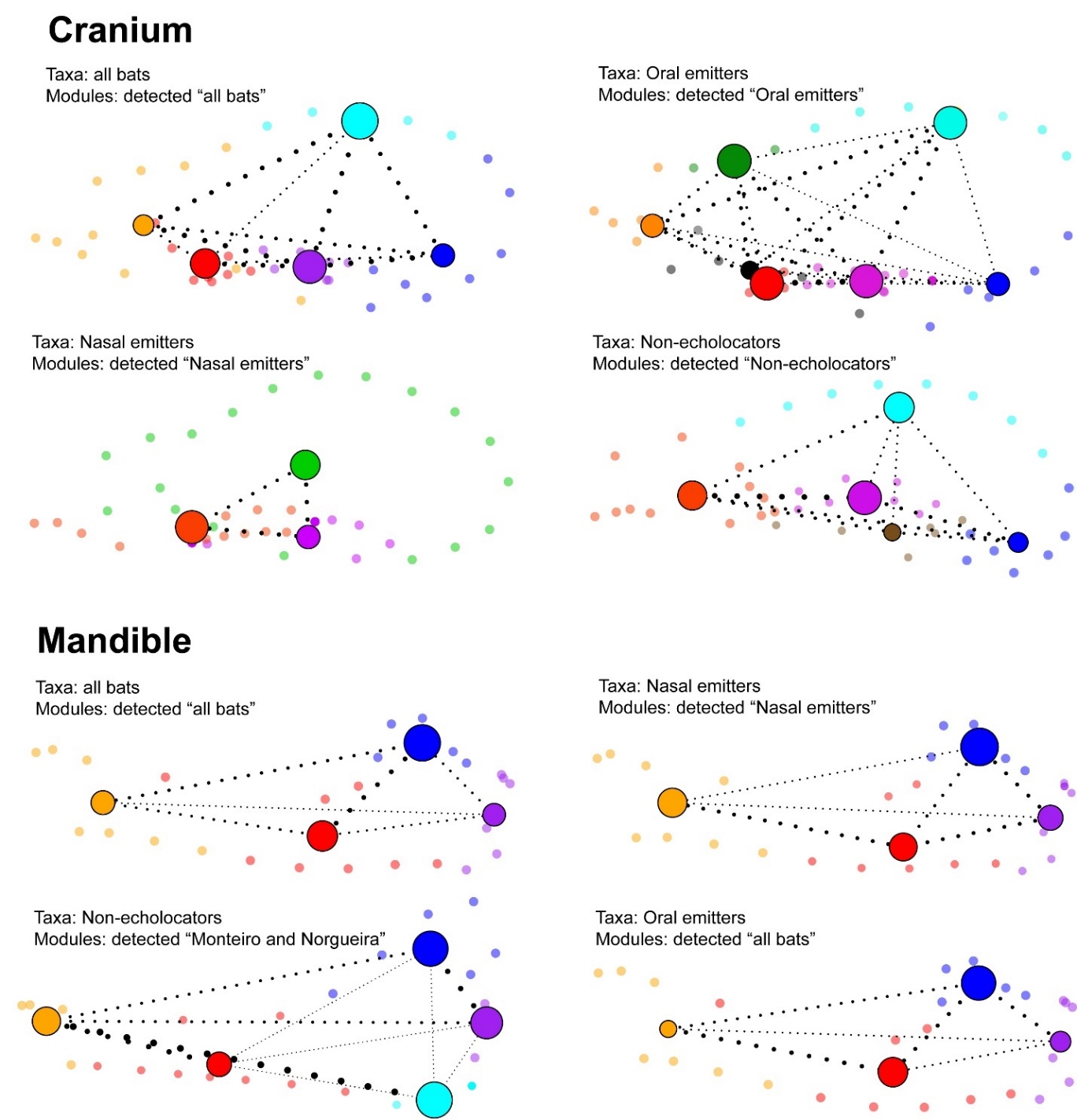


Fig. S9: Results of EMMLi analysis showing the between and within module covariation for each of the best supported modules in each dataset. Small circles show the position of the consensus landmark configurations per dataset. Large circles are the centroid of each module and are scaled by the correlation of landmarks within each module. Dotted lines connecting modules are scaled by the strength of the correlation between modules (thicker lines = stronger correlations). Taxa = which species were included in each analyses – all bats or those possessing a given mode of echolocation. Modules = which modules are illustrated by the colors, either those detected by cluster analyses for a given set of taxa (see Fig. 2 and 3) or one of the three alternative hypotheses examined (Goswami-6 or face/braincase for the cranium, and Monteiro-Norgueira-5 for the mandible).

Table S3: EMMLi results summary for the cranium. Bat refers to modules detected across the full dataset of all species, oral refers to just the oral-emitter dataset, nasal to the nasal-emitter dataset and non to the non-echolocator dataset.

| all bats | modules | within module correlations/ between module correlations | MaxL | K | n | AICc | dAICc |
| --- | --- | --- | --- | --- | --- | --- | --- |
|  | none | NA | -11232.4 | 2 | 861 | 22468.85 | 10135.56 |
|  | bat modules | same/same | -8364.81 | 3 | 861 | 16735.65 | 4402.352 |
|  | bat modules | separate/same | -7038.69 | 7 | 861 | 14091.51 | 1758.214 |
|  | bat modules | same/separate | -7476.45 | 12 | 861 | 14977.26 | 2643.965 |
|  | **bat modules** | **separate/separate** | **-6150.33** | **16** | **861** | **12333.29** | **0** |
|  | face-braincase | same/same | -10337 | 3 | 861 | 20680.04 | 8346.744 |
|  | face-braincase | separate/same | -10337 | 4 | 861 | 20682.06 | 8348.763 |
|  | Goswami 6 Mammal modules | same/same | -8673.07 | 3 | 861 | 17352.16 | 5018.865 |
|  | Goswami 6 Mammal modules | separate/same | -8115.29 | 8 | 861 | 16246.75 | 3913.457 |
|  | Goswami 6 Mammal modules | same/separate | -8055.37 | 17 | 861 | 16145.46 | 3812.17 |
|  | Goswami 6 Mammal modules | separate/separate | -7497.6 | 22 | 861 | 15040.4 | 2707.103 |
| Non echolocators | none | NA | -82.0351 | 2 | 861 | 168.0841 | 1502.133 |
|  | bat modules | same/same | 108.0183 | 3 | 861 | -210.009 | 1124.04 |
|  | bat modules | separate/same | 221.5883 | 7 | 861 | -429.045 | 905.0036 |
|  | bat modules | same/separate | 232.901 | 12 | 861 | -441.434 | 892.6149 |
|  | bat modules | separate/separate | 346.4709 | 16 | 861 | -660.297 | 673.7516 |
|  | non modules | same/same | 457.6275 | 3 | 861 | -909.227 | 424.8219 |
|  | non modules | separate/same | 573.0929 | 7 | 861 | -1132.05 | 201.9944 |
|  | non modules | same/separate | 567.8814 | 12 | 861 | -1111.39 | 222.6541 |
|  | **non modules** | **separate/separate** | **683.3467** | **16** | **861** | **-1334.05** | **0** |
|  | face-braincase | same/same | 81.05105 | 3 | 861 | -156.074 | 1177.975 |
|  | face-braincase | separate/same | 164.9292 | 4 | 861 | -321.812 | 1012.237 |
|  | Goswami 6 Mammal modules | same/same | 198.5419 | 3 | 861 | -391.056 | 942.993 |
|  | Goswami 6 Mammal modules | separate/same | 249.5412 | 8 | 861 | -482.913 | 851.1355 |
|  | Goswami 6 Mammal modules | same/separate | 344.9201 | 17 | 861 | -655.114 | 678.9346 |
|  | Goswami 6 Mammal modules | separate/separate | 395.9194 | 22 | 861 | -746.631 | 587.4178 |
| Oral-emitters | none | NA | -3153.89 | 2 | 861 | 6311.797 | 5957.766 |
|  | bat modules | same/same | -2155.75 | 3 | 861 | 4317.532 | 3963.501 |
|  | bat modules | separate/same | -1059.86 | 7 | 861 | 2133.852 | 1779.821 |
|  | bat modules | same/separate | -1883.94 | 12 | 861 | 3792.238 | 3438.207 |
|  | bat modules | separate/separate | -788.044 | 16 | 861 | 1608.732 | 1254.701 |
|  | oral modules | same/same | -932.323 | 3 | 861 | 1870.674 | 1516.643 |
|  | oral modules | separate/same | -589.809 | 9 | 861 | 1197.83 | 843.7987 |
|  | oral modules | same/separate | -489.483 | 23 | 861 | 1026.284 | 672.253 |
|  | **oral modules** | **separate/separate** | **-146.969** | **29** | **861** | **354.031** | **0** |
|  | face-braincase | same/same | -2970.94 | 3 | 861 | 5947.918 | 5593.887 |
|  | face-braincase | separate/same | -2952.62 | 4 | 861 | 5913.29 | 5559.26 |
|  | Goswami 6 Mammal modules | same/same | -1436.69 | 3 | 861 | 2879.401 | 2525.37 |
|  | Goswami 6 Mammal modules | separate/same | -1153.48 | 8 | 861 | 2323.128 | 1969.097 |
|  | Goswami 6 Mammal modules | same/separate | -1272.12 | 17 | 861 | 2578.968 | 2224.937 |
|  | Goswami 6 Mammal modules | separate/separate | -988.914 | 22 | 861 | 2023.035 | 1669.004 |
| Nasal-emitters | none | NA | -5545.7 | 2 | 861 | 11095.42 | 5837.052 |
|  | bat modules | same/same | -4831.77 | 3 | 861 | 9669.574 | 4411.208 |
|  | bat modules | separate/same | -4335.63 | 7 | 861 | 8685.394 | 3427.027 |
|  | bat modules | same/separate | -4327.34 | 12 | 861 | 8679.051 | 3420.685 |
|  | bat modules | separate/separate | -3831.2 | 16 | 861 | 7695.045 | 2436.678 |
|  | nasal modules | same/same | -2922.41 | 3 | 861 | 5850.857 | 592.4901 |
|  | nasal modules | separate/same | -2669.42 | 5 | 861 | 5348.9 | 90.53314 |
|  | nasal modules | same/separate | -2875.12 | 5 | 861 | 5760.305 | 501.938 |
|  | **nasal modules** | **separate/separate** | **-2622.12** | **7** | **861** | **5258.367** | **0** |
|  | face-braincase | same/same | -4650.08 | 3 | 861 | 9306.191 | 4047.824 |
|  | face-braincase | separate/same | -4513.99 | 4 | 861 | 9036.019 | 3777.652 |
|  | Goswami 6 Mammal modules | same/same | -4803.27 | 3 | 861 | 9612.558 | 4354.192 |
|  | Goswami 6 Mammal modules | separate/same | -3974.87 | 8 | 861 | 7965.917 | 2707.55 |
|  | Goswami 6 Mammal modules | same/separate | -4365.13 | 17 | 861 | 8764.989 | 3506.623 |
|  | Goswami 6 Mammal modules | separate/separate | -3536.74 | 22 | 861 | 7118.688 | 1860.322 |

Table S4: EMMli summary results for the mandible. Bat refers to modules detected across the full dataset of all species, oral refers to just the oral-emitter dataset, nasal to the nasal-emitter dataset and non to the non-echolocator dataset.

|  | Modules | within module correlations/ between module correlations | MaxL | K | n | AICc | dAICc |
| --- | --- | --- | --- | --- | --- | --- | --- |
| all bats | none | No.modules.default | -4414.05 | 2 | 325 | 8832.127 | 5528.473 |
|  | bat modules | same/same | -2346.28 | 3 | 325 | 4698.629 | 1394.975 |
|  | bat modules | separate/same | -1845.98 | 6 | 325 | 3704.221 | 400.5666 |
|  | bat modules | same/separate | -2140.7 | 8 | 325 | 4297.864 | 994.2099 |
|  | **bat modules** | **separate/separate** | **-1640.41** | **11** | **325** | **3303.654** | **0** |
|  | Monteiro and Nogueria | same/same | -2711.23 | 3 | 325 | 5428.533 | 2124.879 |
|  | Monteiro and Nogueria | separate/same | -2585.34 | 7 | 325 | 5185.033 | 1881.379 |
|  | Monteiro and Nogueria | same/separate | -2327.84 | 12 | 325 | 4680.686 | 1377.032 |
|  | Monteiro and Nogueria | separate/separate | -2201.95 | 16 | 325 | 4437.674 | 1134.021 |
| Non echolocators | None | No.modules.default | -235.969 | 2 | 325 | 475.9754 | 575.1389 |
|  | bat modules | same/same | -135.274 | 3 | 325 | 276.6218 | 375.7854 |
|  | bat modules | separate/same | -70.0134 | 6 | 325 | 152.2909 | 251.4544 |
|  | bat modules | same/separate | -114.075 | 8 | 325 | 244.6059 | 343.7694 |
|  | bat modules | separate/separate | -48.8149 | 11 | 325 | 120.4733 | 219.6368 |
|  | non modules | same/same | -86.7537 | 3 | 325 | 179.5822 | 278.7457 |
|  | non modules | separate/same | -18.7557 | 5 | 325 | 47.69947 | 146.863 |
|  | non modules | same/separate | -60.4555 | 5 | 325 | 131.0991 | 230.2626 |
|  | non modules | separate/separate | 7.542522 | 7 | 325 | -0.73173 | 98.43177 |
|  | Monteiro and Nogueria 5 | same/same | -65.0433 | 3 | 325 | 136.1613 | 235.3248 |
|  | Monteiro and Nogueria 5 | separate/same | -7.38995 | 7 | 325 | 29.1332 | 128.2967 |
|  | Monteiro and Nogueria 5 | same/separate | 8.811544 | 12 | 325 | 7.376913 | 106.5404 |
|  | **Monteiro and Nogueria** | separate/separate | **66.46487** | **16** | **325** | **-99.1635** | **0** |
| Oral-emitters | none | No.modules.default | -1224 | 2 | 325 | 2452.046 | 1708.478 |
|  | bat modules | same/same | -707.211 | 3 | 325 | 1420.496 | 676.9275 |
|  | bat modules | separate/same | -466.975 | 6 | 325 | 946.2141 | 202.6457 |
|  | bat modules | same/separate | -600.598 | 8 | 325 | 1217.652 | 474.0835 |
|  | **bat modules** | **separate/separate** | **-360.363** | **11** | **325** | **743.5685** | **0** |
|  | oral modules | same/same | -652.888 | 3 | 325 | 1311.852 | 568.283 |
|  | oral modules | separate/same | -428.564 | 6 | 325 | 869.3923 | 125.8238 |
|  | oral modules | same/separate | -585.805 | 8 | 325 | 1188.066 | 444.4974 |
|  | oral modules | separate/separate | -361.481 | 11 | 325 | 745.805 | 2.236558 |
|  | Monteiro and Nogueria 5 | same/same | -647.862 | 3 | 325 | 1301.798 | 558.2292 |
|  | Monteiro and Nogueria 5 | separate/same | -563.721 | 7 | 325 | 1141.796 | 398.2275 |
|  | Monteiro and Nogueria 5 | same/separate | -548.7 | 12 | 325 | 1122.399 | 378.8308 |
|  | Monteiro and Nogueria 5 | separate/separate | -464.56 | 16 | 325 | 962.8852 | 219.3168 |
| Nasal-emitters | none | No.modules.default | -1563.95 | 2 | 325 | 3131.936 | 2484.341 |
|  | bat modules | same/same | -786.878 | 3 | 325 | 1579.831 | 932.2361 |
|  | bat modules | separate/same | -529.088 | 6 | 325 | 1070.44 | 422.8452 |
|  | bat modules | same/separate | -678.663 | 8 | 325 | 1373.781 | 726.1863 |
|  | bat modules | separate/separate | -420.873 | 11 | 325 | 864.5888 | 216.9938 |
|  | nasal modules | same/same | -605.848 | 3 | 325 | 1217.771 | 570.1759 |
|  | nasal modules | separate/same | -390.444 | 6 | 325 | 793.153 | 145.5581 |
|  | nasal modules | same/separate | -527.779 | 8 | 325 | 1072.014 | 424.4195 |
|  | **nasal modules** | **separate/separate** | **-312.376** | **11** | **325** | **647.5949** | **0** |
|  | Monteiro and Nogueria 5 | same/same | -909.871 | 3 | 325 | 1825.817 | 1178.222 |
|  | Monteiro and Nogueria 5 | separate/same | -769.192 | 7 | 325 | 1552.737 | 905.1418 |
|  | Monteiro and Nogueria 5 | same/separate | -741.299 | 12 | 325 | 1507.597 | 860.002 |
|  | Monteiro and Nogueria 5 | separate/separate | -600.619 | 16 | 325 | 1235.004 | 587.4093 |

Modularity in Evolutionary Rates

Table S5: Evolutionary rate heterogeneity of the bat cranium and mandible using the σ^2^_mult_ statistic of Adams 2014. Ratio gives the comparison between the single highest and lowest evolutionary rates within each model. * indicates statistical significance after Holm-Bonferroni correction.

|  | Cranium |  |  |  | Mandible |  |  |
| --- | --- | --- | --- | --- | --- | --- | --- |
|  | **Modules** | **Number of Rates** | **ratio** | **p** | **Number of Rates** | **ratio** | **p** |
| All bats | All bats | 5 | 2.03 | 0.443 | 5 | 1.46 | 0.432 |
|  | None | 42 | 4.4382 | 0.003* | 26 | 4.28 | 0.001* |
|  |  |  |  |  |  |  |  |
| non-echolocators | All bats | 5 | 2.2907 | 0.879 | 4 bats | 2.0966 | 0.092 |
|  | None | 42 | 6.7151 | 0.008 | 26 | 6.7041 | 0.004* |
|  | Non-echolocators | 5 | 2.0971 | 0.989 | 3 | 1.4287 | 0.618 |
| oral echolocators | All bats | 5 | 2.7892 | 0.003* | 4 | 1.6648 | 0.129 |
|  | None | 42 | 6.7748 | 0.003* | 26 | 4.726 | 0.001* |
|  | Oral emitters | 7 | 3.7105 | 0.355 | 4 | 1.8049 | 0.074 |
| nasal echolocators | All bats | 5 | 2.0155 | 0.708 | 4 | 1.5482 | 0.651 |
|  | None | 42 | 4.334 | 0.04 | 25 | 3.7282 | 0.01 |
|  | Nasal emitters | 3 | 1.0733 | 1 | 4 | 1.55 | 0.837 |

Table S6: Examination of evolutionary rate heterogeneity across the mandible and cranium in bats based on the likelihood approach described herein. Models included all varying rates (all), equal rates (one), and modular rates based on modules detected across all bats (Mod-Bats) or within each echolocator type (Mod-Echo). Likelihoods were calculated for the single rate and modular rate modules using a constrained R matrix either including or excluding trait covariation. Ratio gives the comparison of the single highest and lowest evolutionary rates per model, and is equal to that provided in Table 5.

|  |  |  | Cranium | |  | |  |  | Mandible | |  |  |
| --- | --- | --- | --- | --- | --- | --- | --- | --- | --- | --- | --- | --- |
|  |  |  | **No Covariation** | | **With Covariation** | |  |  | **No Covariation** | | **With Covariation** |  |
|  | **# rates** | **ratio** | **Lik** | **ΔAIC** | **Lik** | **ΔAIC** | **# rates** | **ratio** | **Lik** | **ΔAIC** | **Lik** | **ΔAIC** |
| all bats |  |  |  |  |  |  |  |  |  |  |  |  |
| All | 42 | 4.438218 | **26122** | **0** | **32544** | **0** | 26 | 4.279 | 14759 | 0 | 19269.1 | 0 |
| Mod | 5 | 2.034068 | 25915.3 | 338.62 | 32103 | 807.22 | 4 | 1.4603 | 14649 | 175.86 | 18902.3 | 689.47 |
| One | 1 | 1 | 25705.8 | 749.73 | 31900 | 1206.3 | 1 | 1 | 14610 | 247.08 | 18851.5 | 785.03 |
|  |  |  |  |  |  |  |  |  |  |  |  |  |
| Non-echolocators | | |  |  |  |  |  |  |  |  |  |  |
| All | 42 | 6.715087 | **3987.7** | **0** | **5034** | **0** | 26 | 6.7041 | 2186.1 | 0.865 | **2952.33** | **0** |
| Mod-Bats | 5 | 2.290707 | 3953.66 | 292.004 | 4822 | 351.014 | 4 | 2.0966 | **2164.5** | **0** | 2683.09 | 494.48 |
| Mod-Echo | 5 | 2.097091 | 3953.88 | 293.585 | 4835 | 324.345 | 3 | 1.4287 | 2154.8 | 17.481 | 2631.74 | 595.18 |
| One | 1 | 1 | 3926.42 | 340.499 | 4777 | 432.418 | 1 | 1 | 2150.3 | 22.442 | 2713.9 | 426.87 |
| Oral-Emitters | |  |  |  |  |  |  |  |  |  |  |  |
| All | 42 | 6.774761 | **11695** | **0** | **14607** | **0** | 26 | 4.726 | **6935.9** | **0** | **8711.64** | **0** |
| Mod-Bats | 5 | 2.789193 | 11596.8 | 120.75 | 14319 | 501.93 | 4 | 1.6648 | 6875.7 | 76.42 | 8542.94 | 293.4 |
| Mod-Echo | 7 | 3.71054 | 11601.8 | 116.74 | 14316 | 512.6 | 4 | 1.8049 | 6882.1 | 63.49 | 8497.46 | 384.36 |
| One | 1 | 1 | 11438.3 | 431.87 | 14147 | 837.24 | 1 | 1 | 6849.7 | 122.34 | 8499.79 | 373.71 |
| Nasal-Emitters | |  |  |  |  |  |  |  |  |  |  |  |
| All | 42 | 4.334035 | **11047** | **0** | **14011** | **0** | 26 | 3.728 | **5928.5** | **0** | **7997.74** | **0** |
| Mod-Bats | 5 | 2.015462 | 10978.8 | 60.02 | 13779 | 390.94 | 4 | 1.548 | 5888.4 | 36.14 | 7823.28 | 298.17 |
| Mod-Echo | 3 | 1.794342 | 10957.3 | 101.06 | 13793 | 358.66 | 4 | 1.55 | 5888.2 | 36.44 | 7826.66 | 298.17 |
| One | 1 | 1 | 10908.3 | 195.03 | 13742 | 455.69 | 1 | 1 | 5873.9 | 59.2 | 7817.15 | 311.18 |
